# A mouse model for Li-Fraumeni-Like Syndrome with cardiac angiosarcomas associated to *POT1* mutations

**DOI:** 10.1371/journal.pgen.1010260

**Published:** 2022-06-21

**Authors:** Paula Martínez, Raúl Sánchez-Vázquez, Iole Ferrara-Romeo, Rosa Serrano, Juana M. Flores, Maria A. Blasco

**Affiliations:** 1 Telomeres and Telomerase Group, Molecular Oncology Program, Spanish National Cancer Centre (CNIO), Madrid, Spain; 2 Animal Surgery and Medicine Department, Faculty of Veterinary Science, Complutense University of Madrid, Madrid, Spain; Seattle Children’s Research Institute, UNITED STATES

## Abstract

The shelterin protein POT1 has been found mutated in many different familial and sporadic cancers, however, no mouse models to understand the pathobiology of these mutations have been developed so far. To address the molecular mechanisms underlying the tumorigenic effects of POT1 mutant proteins in humans, we have generated a mouse model for the human *POT1*^*R117C*^ mutation found in Li-Fraumeni-Like families with cases of cardiac angiosarcoma by introducing this mutation in the *Pot1a* endogenous locus, *knock-in* for *Pot1a*^*R117C*^. We find here that both mouse embryonic fibroblasts (MEFs) and tissues from *Pot1a*^+/*ki*^ mice show longer telomeres than wild-type controls. Longer telomeres in *Pot1a*^+/*ki*^ MEFs are dependent on telomerase activity as they are not found in double mutant *Pot1a*^+/*ki*^ *Tert*^*-/-*^ telomerase-deficient MEFs. By using complementation assays we further show that POT1a pR117C exerts dominant-negative effects at telomeres. As in human Li-Fraumeni patients, heterozygous *Pot1a*^+/*ki*^ mice spontaneously develop a high incidence of angiosarcomas, including cardiac angiosarcomas, and this is associated to the presence of abnormally long telomeres in endothelial cells as well as in the tumors. The *Pot1a*^*+/R117C*^ mouse model constitutes a useful tool to understand human cancers initiated by *POT1* mutations.

## Author summary

We have generated a mouse model for the human *POT1*^*R117C*^ mutation found in Li-Fraumeni-Like (LFL) families with cases of cardiac angiosarcoma by introducing this mutation in the *Pot1a* endogenous locus, *knock-in* for *Pot1a*^*R117C*^. The *Pot1a*^+/*ki*^ mice show longer telomeres than wild-type controls. Longer telomeres in mutant mice are dependent on telomerase activity as they are not found in a telomerase deficient background. As in human Li-Fraumeni patients, heterozygous *Pot1a*^+/*ki*^ mice spontaneously develop a high incidence of angiosarcomas, including cardiac angiosarcomas, and this is associated to the presence of abnormally long telomeres in endothelial cells as well as in the tumors. The *ki-Pot1a* ^*R117C*^ mouse constitutes a potential pre-clinical mouse model for LFL syndrome presenting with high angiosarcoma incidence that could provide in the future a very useful tool for the study of treatments for these tumors.

## Introduction

Telomeres are protective structures at the ends of chromosomes essential to ensure chromosome stability [1,2]. Vertebrate telomeres consist of tandem repeats of the TTAGGG DNA sequence bound by a six-protein complex known as shelterin, which protects telomeres from chromosomal aberrations and the activation of a persistent DNA damage response (DDR), as well as regulates telomerase activity at chromosome ends [1–3]. The shelterin complex is formed by TRF1, TRF2, RAP1, POT1, TIN2 and TPP1 [1,2]. Telomerase elongates telomeres at the pluripotent stage but is silenced in the majority of cell types after birth leading to progressive telomere loss, a hallmark of ageing [4]. Telomerase encompasses a reverse transcriptase catalytic subunit (TERT) and an RNA template (Terc), which recognizes the hydroxyl group (OH) at the 3′ end of the G-strand overhang and elongates the telomere [5].

Telomere maintenance is a hallmark of cancer cells as it allows for indefinite cell division capability [6]. To avoid telomere loss associated to cell division, the vast majority of cancers reactivate the telomere-elongating enzyme telomerase [5,7]. Indeed, telomerase is a highly mutated gene in many different cancer types [8–10]. Telomerase is not the only telomere component altered in cancer. More recently, mutations in *POT1*, the gene encoding for a component of the shelterin telomere protective complex, have been identified in several types of human sporadic or familial cancers. These include mantle cell lymphoma, adult T-cell leukemia/lymphoma, parathyroid adenoma, colorectal cancer, pulmonary sarcomatoid carcinoma, sporadic and familial chronic lymphocytic leukemia (CLL), familial melanoma, glioma, thyroid cancer and Li-Fraumeni like- (LFL) syndrome families [11–26]. We and others have shown that *POT1* mutations found in CLL, melanoma, Hodgkin lymphoma, T-cell lymphoma and LFL syndrome result in longer telomeres and are associated with higher chromosomal instability [13,16,18,23,27,28], another hallmark of cancer [6]. In particular, we demonstrated that families with Li-Fraumeni Like Syndrome carrying POT1 mutations showed increased incidence of different tumor types including the rare cardiac angiosarcoma (CAS) [13,29]. Initially, a single mutation was found in these LFL families, p.(R117C), which was also different from the *POT1* mutations found in other cancer types. The wide-tumor spectrum shown in these LFL families made this *POT1* mutation especially interesting since it could be at the origin of very different tumor types. Of particular interest, CAS is a rare and infrequent tumor with very bad prognosis with unknown pathogenesis and in need of new therapies. Generating mouse models carrying LFL-associated POT1 mutations will be instrumental to understand the role of POT1 in the pathobiology of various cancers and to search for new therapeutic opportunities. Humanized mouse models for these diseases are lacking.

Both POT1 and its interacting protein TPP1 have been shown to regulate telomerase activity at chromosome ends [30–33]. While human cells contain only one *POT1* gene, mouse cells have *Pot1a* and *Pot1b* [34]. The two mouse POT1 proteins are highly homologous and can associate with telomeric DNA but while the *Pot1b* knock-out (KO) mouse is viable and does not present phenotypes except when combined with telomerase deficiency [35,36], *Pot1a* deletion in mice is embryonic lethal hampering the study of POT1a role in cancer development [34].

To address the mechanisms underlying the tumorigenic effects of POT1 mutant proteins in humans, we have generated a mouse model for the human *POT1*^*R117C*^ mutation found in LFL families with cases of CAS by introducing this mutation in the *Pot1a* endogenous locus, *knock-in* for *Pot1a*^*R117C*^, thus generating *Pot1a*^*ki*^ mice. Homozygous *Pot1a*^*ki*/*ki*^ are embryonic lethal while heterozygous *Pot1a*^+/*ki*^ mice are viable and show longer telomeres in proliferative tissues, i.e. whole blood cells, bone marrow and endothelia. The POT1a p.R117C-mediated telomere lengthening was dependent on the presence of telomerase activity in MEFs, as it did not occur in the context of telomerase-deficient *Tert*^*-/-*^ mice. The *Pot1a*^+/*ki*^ mice spontaneously develop angiosarcomas, including cardiac angiosarcomas, at higher frequency than wild-type mice. The *knock-in Pot1a*^*R117C/+*^ mouse model constitutes a useful tool to understand human cancers initiated by POT1 mutation.

## Results

### Embryonic lethality in mice carrying the Li-Fraumeni-like mutation *Pot1a*^*R117C/R117C*^

**Fig 1:**
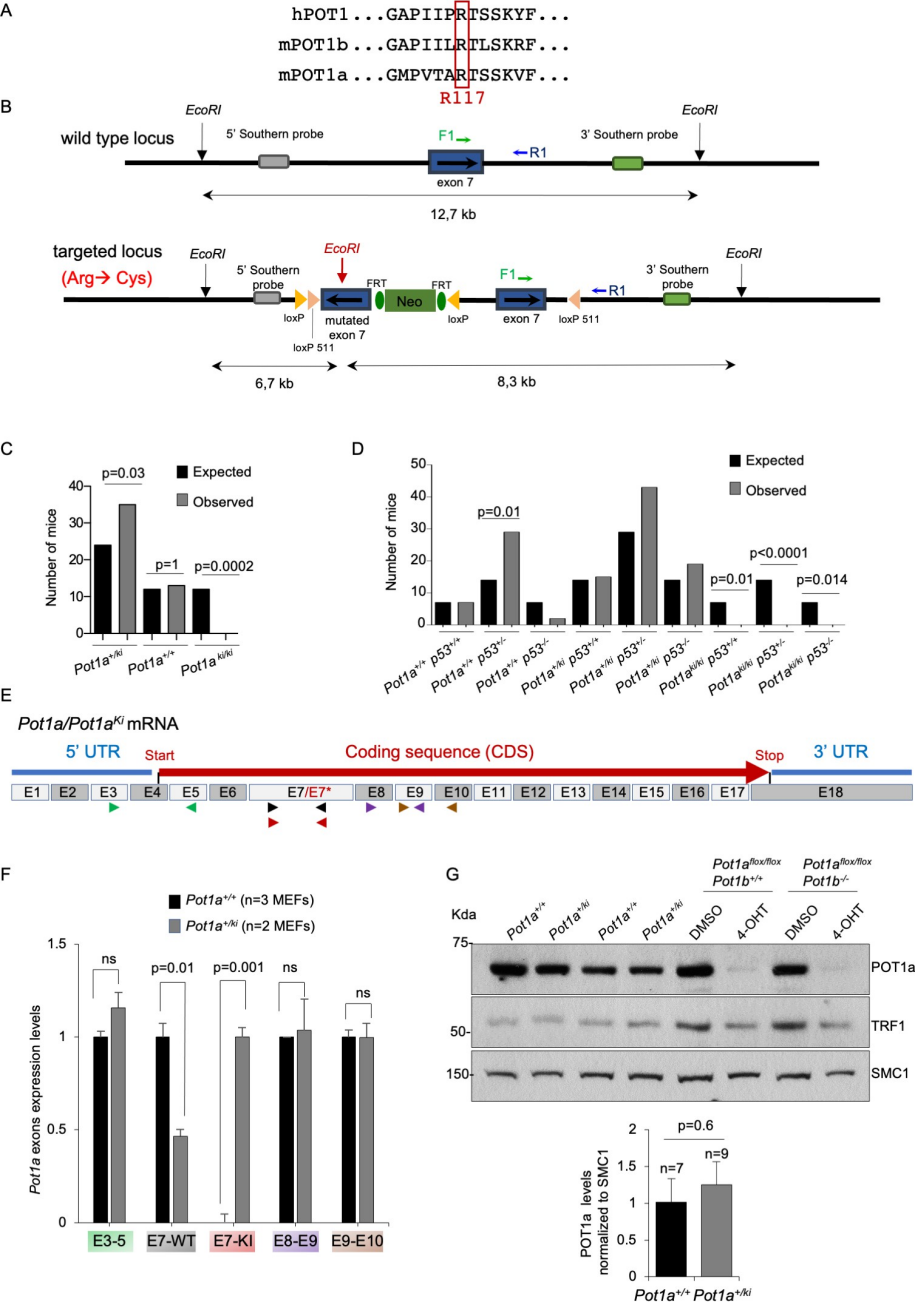
Embryonic lethality of *Pot1a*^*R117C/R117C*^ (*Pot1a*^*ki/ki*^) mice is not rescued by p53 deficiency. **A.** Alignment among human POT1, mouse POT1b and mouse POT1a from residue 111 to 123 in the three proteins. The conserved R117 is indicated. **B.** Schematic representation of *Pot1a* wild-type and targeted locus. The loxP, loxP511 and FRT sites are indicated. The genomic location of the 5’- and 3’- probes for southern analysis are drawn. Genomic DNA was digested with EcoR1 restriction enzymes for southern analysis and the size of the fragments are indicated. **C-D.** Expected and observed number of mice of the offspring from *Pot1a*^*+/ki*^ (C) and *Pot1a*^*+/ki*^ *p53*^*+/-*^ (D) intercrosses. The Fisher’s exact test was used to determine statistical significance. p-values are indicated**. E.** Schematic representation of *Pot1a* and *Pot1a*^*ki*^ mRNA. The R117C substitution is within exon 7 (E7*). Five different primers pairs were used to quantify transcripts levels corresponding to E3-E5 (green arrows), E7 (black and red arrows for E7 wildtype and E7*, respectively), E8-E9 (purple arrows) and E9-E10 (brown arrows). The scheme is not drawn to scale. **F.** Quantitative qRT-PCR analysis of *Pot1a* and *Pot1a*^*ki*^ mRNA levels in *Pot1a*^*+/+*^ and *Pot1a*^*+/ki*^ MEFs. mRNA expression levels were normalized to wild-type. **G.** Representative Western blot images and quantification of POT1a and TRF1 protein levels in *Pot1a*^*+/+*^ and *Pot1a*^*+/ki*^ MEFs. *Pot1a*^*flox/flox*^ *Pot1b*^*+/+*^ and *Pot1a*^*flox/flox*^ *Pot1b*^*-/-*^ MEFs grown with or without 4-Hydroxytamoxifen (4-OHT) for six days were used as control for specificity of POT1a antibody. SMC1 was used as loading control. A t-test two tailed was used for statistical analysis. The p-value is indicated. Mean values +/- SEM are represented. The number of MEFs analyzed per genotype is indicated.

To understand the role of POT1 in the pathobiology of Li-Fraumeni-Like (LFL) syndrome, here we set to generate a *knock-in* mouse harboring the mouse equivalent to the *POT1*^*R117C*^ mutation found in LFL families [13]. In the mouse there are two *Pot1* genes, *Pot1a* and *Pot1b*, however, only deletion of *Pot1a* results in embryonic lethality [34,37], suggesting that *Pot1a* is the essential shelterin gene analogous to the single human *POT1* gene. Residue R117 in human POT1 is conserved in mouse POT1a and POT1b at the same position (**Fig 1A**). Thus, we generated a *Pot1a knock-in* allele carrying the same missense Fmutation, *Pot1a*^*R117C*^ found in human LFL families (**Figs 1B** and **S1A–S1F**; Materials and Methods). Intercrosses between heterozygous mice carrying the *Pot1a*^*+/flexR117C*^ allele, referred here as *Pot1a*^*+/flex*^ mice, failed to render viable homozygous *Pot1a*^*flex/flex*^ offspring indicating embryonic lethality of the *Pot1a*^*flex*^ allele when in homozygosis (**S2A Fig**). In agreement with early embryonic lethality of the conditional allele, we could not derive mouse embryonic fibroblasts (MEFs) of the *Pot1a*^*flex/flex*^ genotype (E.11.5) from intercrosses between *Pot1a*^*+/flex*^ parental mice (**S2B Fig**).

*Pot1a*^*+/flex*^ heterozygous mice were crossed with transgenic mice expressing the Cre recombinase under the control of the adenovirus EIIa promoter, which targets expression of the Cre recombinase to the early stages of embryonic development, oocytes, and preimplantation embryos [38] to generate heterozygous *Pot1a*^*+/ki-R117C*^ mice, referred here as *Pot1a*^*+/ki*^ mice. Expression of the Cre recombinase leads to replacement of the wild-type exon7 for the mutated exon harbouring the R117C missense mutation (**Fig 1B**). Intercrosses of *Pot1a*^*+/ki*^ heterozygous mice, however, did not render any *Pot1a*^*ki/ki*^ offspring indicating embryonic lethality of the mutant *Pot1a*^*ki*^ allele when in homozygosis (**Fig 1C**). We have previously described that some phenotypes associated to deletion of shelterin components, can be rescued by simultaneous deletion of p53 [33,39], thus we set to generate *Pot1a*^*ki/ki*^ *p53*^*-/-*^ double mutant mice to rescue lethality. However, p53 deficiency did not rescue the lethality associated to the *Pot1a*^*ki*^ allele when in homozygosis (**Fig 1D**). In agreement with embryonic lethality, we also failed to obtain *Pot1a*^*ki/ki*^ *p53*^*-/-*^ double mutant MEFs (E.11.5 and E13.5) (**S2C Fig**). Thus, we set to study the effects of the mutant allele when in heterozygosis.

To understand the lethality associated to the *Pot1a* mutant allele, we analyzed the transcriptional expression of different *Pot1a* exons in *Pot1a*^*+/+*^ and *Pot1a*^*+/ki*^ MEFs (**Fig 1E, 1F** and **S1 Table**). We found that the *Pot1a*^*ki*^ allele is expressed to similar levels than the *Pot1a*^*+*^ allele in exons 3 to 5, exon 8 to 9 and exon 9 to 10 in *Pot1a*^*+/ki*^ and *Pot1a*^*+/+*^ MEFs (**Fig 1E and 1F**). As expected, the mutated exon 7 (E7*) is only detected in *Pot1*^*+/ki*^ MEFs and the wild-type exon 7 (E7) is reduced by 50% in *Pot1a*^*+/ki*^ MEFs compared to *Pot1a*^*+/+*^ controls (**Fig 1F**). We confirmed these results *in vivo* in the tail of *Pot1a*^*+/+*^, *Pot1a*^*+/flex*^ and *Pot1a*^*+/ki*^ mice (**S2D and S2E Fig**). Of note, mutant exon 7 was expressed to similar levels in *Pot1a*^*+/flex*^ and *Pot1a*^*+/ki*^ mice, indicating that intron 6 is not properly processed in *Pot1a*^*+/flex*^ mice, thus providing an explanation for the embryonic lethality observed in *Pot1a*^*flex/flex*^ mice which behaves as a *Pot1a knock-out* allele [34] (**S2A, S2D, and S2E Fig**). In addition, we sequenced the exon 3 to exon 9 PCR amplification products from *Pot1a*^*+/+*^ and *Pot1a*^*+/ki*^ MEFs cDNA (**S1 Table**). While the sequencing reaction from *Pot1a*^*+/+*^ cDNA rendered a single spectrum in the region corresponding to exon7, a double spectrum was observed in the reaction from *Pot1a*^*+/ki*^ cDNA, confirming that both *Pot1a*^*+*^ and *Pot1*^*ki*^ alleles are transcribed (**S3A and S3B Fig**).

To confirm correct expression of the mutant POT1a protein, we analyzed POT1a protein levels by western blot in nuclear extracts from of *Pot1a*^*+/+*^ and *Pot1a*^*+/ki*^ MEFs using a homemade monoclonal rat antibody specific for POT1a (Clone name POP148C, CNIO Monoclonal Core Unit catalogue, www.cnio.es) No differences in total POT1a protein levels were detected between *Pot1a*^*+/+*^ and *Pot1a*^*+/ki*^ MEFs, indicating that the mutant POT1a p.R117C protein is properly transduced and stable (**Fig 1G**). As negative control for antibody specificity, we used MEFs deleted for *Pot1a* and either wild-type (*Pot1a*^*flox/flox*^ *Pot1b*^*+/+*^) or *knock-out* for *Pot1b* (*Pot1a*^*flox/flox*^ *Pot1b*^*-/-*^) (Materials and Methods) (**Fig 1G**). As a positive control, total TRF1 levels were similar in *Pot1a*^*+/ki*^ and wild-type MEFs (**Fig 1G**).

To assay whether the *Pot1a*^*+/ki*^ and wild-type MEFs present different levels of POT1a bound to telomeres and whether expression of POT1a p.R117C was altering the binding of other shelterin components to the telomeres, we next performed a Chromatin Immuno Precipitation (ChIP) with anti-POT1a, anti-TRF1 and anti-TRF2 antibodies (Materials and Methods). We did not detect significant differences in immunoprecipitated telomeric DNA with any of the antibodies used between *Pot1a*^*+/ki*^ *p53*^*-/-*^ and *Pot1b*^*+/+*^ *p53*^*-/-*^ MEFs, suggesting that the mutant POT1a-p.R117C protein is able to bind telomeres and that this binding does not disturb the binding of TRF1 and TRF2 shelterin proteins (**S4A–S4D Fig)**. However, it should be pointed out that in these heterozygous *Pot1a*^*+/ki*^ cells we cannot distinguish between POT1a wild-type and mutant variants. Potential compensatory effects could therefore not be ruled out. In agreement with ChIP data, we found similar TRF1 protein levels in the intestine and liver of *Pot1a*^*+/+*^ and *Pot1a*^*+/ki*^ mice as determined by immunofluorescence with anti-TRF1 antibody (**S4E and S4F Fig**).

### POT1a p.R117C expression leads to slightly higher numbers of TIFs but does not elicit a strong DDR at telomeres

**Fig 2:**
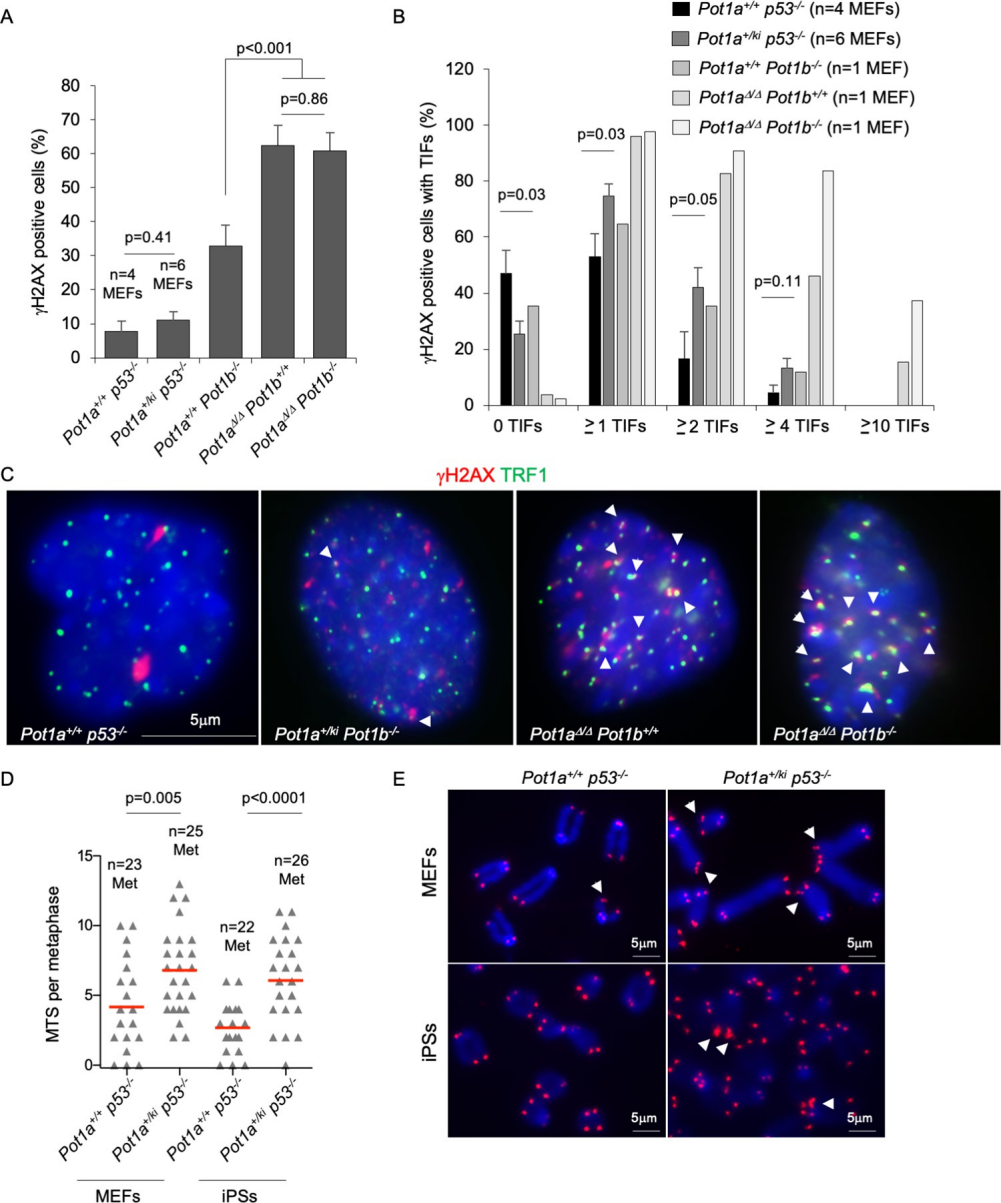
POT1a-R117C expression results in mild increased telomere damage and telomere fragility. **A.** Percentage of γH2AX positive cells. **B.** Percentage of cells of the indicated genotype presenting Telomere-Induced Foci (TIFs). n = number of MEFs. **C.** Representative images of γH2AX and TRF1 immunofluorescence staining. TIFs were detected by γH2AX and TRF1 co-localizing foci (white arrowheads). **D.** Number of multitelomeric signals (MTS) per metaphase in MEFs and iPS cells of the indicated genotype. n = number of metaphases. **E.** Representative Q-FISH images of metaphase spreads in MEFs and iPS cells of the indicated genotypes. White arrowheads indicate MTS. A t-test two tailed was used for statistical analysis. Error bars represent standard error. The p-value is indicated.

POT1a represses the DNA damage checkpoint at telomeres by preventing ATR activation as well as aberrant homologous recombination at telomeres [34,37]. In order to understand the pathobiology of the increased telomere length and increased cancer susceptibility found by us in Li-Fraumeni-Like patients carrying the *POT1*^*R117C*^ mutation [13], we set to study the *in vivo* phenotypes of *Pot1a knock-in* mutant mice. In particular, we previously described that lymphocytes from human *POT1*^*R117C*^ carriers present longer telomeres and higher incidence of multitelomeric signals, a chromosomal aberration associated to increased telomere fragility [13]. Thus, we first analyzed the telomeric phenotypes of MEFs expressing the POT1a-p.R117C protein. Global genomic DNA damage was determined by quantifying the percentage of cells positive for the DNA damage marker γH2AX and telomeric DNA damage was determined by quantification of the so-called telomere induced foci (TIFs), which were detected by immunocolocalization of γH2AX and the TRF1 telomere-binding protein (**Fig 2A–2C**). We did not observe significant differences in global DNA damage between *Pot1a*^*+/ki*^ and *Pot1a*^*+/+*^ MEFs (**Fig 2A**). We found a mild increase in the number of cells presenting 1–2 TIFs in *Pot1a*^*+/ki*^ compared to *Pot1a*^*+/+*^ MEFs (**Fig 2B**). For comparison purposes, we performed similar analysis in inmortalized *Pot1b*, *Pot1a* and in *Pot1a Pot1b* deleted MEFs (**Fig 2A–2C**) (kindly provided by S. Chang). We find that *Pot1a* deletion induces a significant higher increase in the percentage of damaged cells as well as in the number of TIFs compared to *Pot1a*^*+/ki*^ cells (**Fig 2A–2D**), thus suggesting that the *POT1*^*R117C*^ mutation does not induce a strong DNA damage response (DDR) at telomeres.

We next generated induced pluripotent stem cells (iPS cells) from *Pot1a*^*+/ki*^ *p53*^*-/-*^ and *Pot1a*^*+/+*^ *p53*^*-/-*^ MEFs by transduction of the Yamanaka factors, OCT4, SOX2 and KLF4 [40]. To confirm induction of pluripotency, we analyzed the expression of the pluripotent marker nanog, of the telomerase catalytic subunit TERT, as well as of the shelterin protein TRF1, previously shown to be greatly induced during induction of pluripotency (**S5A and S5B Fig**) [41]. In agreement with induction of pluripotency, iPS showed a 8-fold increase in TRF1 protein level, nanog expression, and increased *Tert* mRNA expression compared to the parental MEFs (**S5A and S5B Fig**). Of note, induction of pluripotency and expression of pluripotency markers was not altered in *Pot1a*^*+/ki*^ *p53*^*-/-*^ cells compared to the *Pot1a*^*+/+*^ *p53*^*-/-*^ controls (**S5A and S5B Fig**). Next, we set to address chromosomal aberrations in metaphase spreads from MEFs and iPS cells of both *Pot1a*^*+/ki*^ *p53*^*-/-*^ and *Pot1a*^*+/+*^ *p53*^*-/-*^ genotypes. Occurrence of the so-called “multitelomeric signals” or MTS has been previously associated to increased telomere damage and telomere fragility as the consequence of defective telomere capping [39,42,43]. We found a mild but significant increased abundance of MTS in both MEFs and iPS cells of the *Pot1a*^*+/ki*^ *p53*^*-/-*^ genotype compared to wild-type control cells (**Fig 2D and 2E**).

To address induction of DNA damage associated to the *POT1*^*R117C*^ mutation, we checked the levels of phospho-CHK1 and phospho-RPA in MEFs and iPS cells expressing mutant POT1a p.R1171C protein as a read out of ATR activation (**S6A and S6B Fig**). As positive control for replicative stress, we treated the cells with 2mM hydroxyurea for three hours. In agreement with the low levels of telomeric DNA damage found in *Pot1a*^*+/ki*^ *p53*^*-/-*^ cells (**Fig 2B–2E**), we did not detect either pCHK1 or pRPA in MEF or iPS cells carrying *Pot1a*^*ki*^ allele (**S6A and S6B Fig**).

Altogether, these results indicate that POT1a-p.R117C protein results in alterations in telomere capping structure that are not sufficient to elicit a strong DNA damage response.

### POT1a.R117C mutant protein expression leads to telomere lengthening in a telomerase-dependent manner

**Fig 3:**
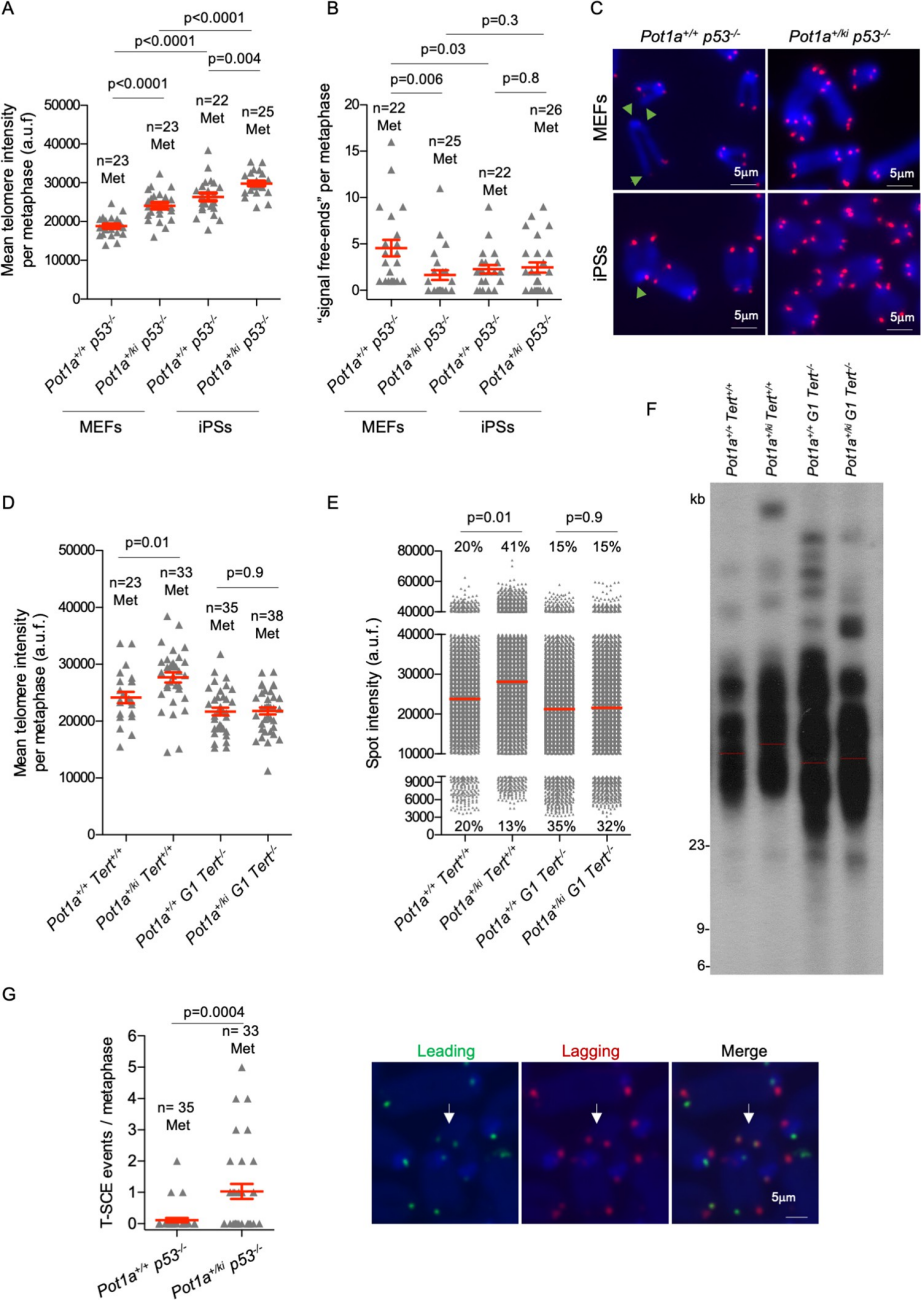
POT1a p.R117C expression results in longer telomeres in a telomerase dependent-manner. **A,B.** Mean telomere intensity (A) and number of signal-free ends (B) per metaphase in MEFs and IPs of the indicated genotype. n = number of metaphases. **C.** Representative Q-FISH images of metaphase spreads in MEFs and iPS cells of the indicated genotypes. Green arrowheads indicate signal free ends. **D,E.** Mean telomere intensity per metaphase (D) and mean spot intensity (E) in MEFs of the indicated genotype. The percentage of short and long telomeres are indicated. The percentage of short and long telomeres was defined as fluorescence intensity below the 20^th^ and above 80^th^ percentile, respectively of the fluorescence intensity values of the wild type. **F.** Telomere restriction fragment (TRF) analysis in MEFs of the indicated genotype. The red line represents median intensity. **G.** Telomeric sister chromatid exchange (T-SCE) events in MEFs of the indicated genotype and a representative CO-FISH images of metaphases hybridized with probes against the leading (green fluorescence) and lagging (red fluorescence) telomere. T-SCE events are indicated with arrows. A T-SCE was considered positive when it was observed with both the leading and lagging strand probes and involved a reciprocal exchange of telomere signal. n = number of metaphases. A t-test two tailed was used for statistical analysis. Error bars represent standard error. The p-values are indicated.

POT1 has been previously shown to have an important role regulation of telomerase activity at chromosome ends [30–33]. Thus, we set to address the telomere length in MEFs and induced pluripotent stem cells (iPS) expressing the POT1a-p.R117C protein. To this end, we performed Quantitative Fluorescence *In Situ* Hybridization (Q-FISH) on metaphase spreads, which allows to measure individual telomere fluorescence signals at each chromosome end as well as the percentage of undetectable telomere signals or “signal-free ends”. We used two independent MEFs from each genotype in a *p53*-null background, *Pot1a*^*+/+*^*p53*^*-/-*^ and *Pot1a*^*+/ki*^*p53*^*-/-*^. Interestingly, mean telomere length per metaphase was significantly higher in *Pot1a*^*ki*^ carriers compared to the *Pot1a* wild-type controls (**Fig 3A**). Importantly, the percentage of “signal-free ends” was also significantly lower in the *Pot1a*^*+/ki*^*p53*^*-/-*^ compared to *Pot1a*^*+/+*^*p53*^*-/-*^ MEFs (**Fig 3B and 3C**). As telomerase has been shown to preferentially elongate the shortest telomeres both in mice and yeast [44,45], these results suggest increased elongation of short telomeres by telomerase as the consequence of the *Pot1a*^*ki*^ mutation in MEFs.

Induction of Pluripotent Stem cells (iPS) from differentiated parental cells leads to a net telomere elongation that is dependent on telomerase activity [46]. To study the effect of the *Pot1a*^*ki*^ mutation in net telomere elongation, we measured telomere length by Q-FISH in iPS cells at passage 10. We observed a similar telomere lengthening in iPS cells of both genotypes compared to the parental MEFs (**Fig 3A**). Accordingly, the percentage of “signal-free ends” was also similar in *Pot1a*^*+/ki*^*p53*^*-/-*^ and the *Pot1a*^*+/+*^*p53*^*-/-*^ controls (**Fig 3B and 3C**), in agreement with elongation of short telomeres as the consequence of telomerase over-expression and increased access of telomerase to the telomeres during induction of pluripotency in the wild-type iPS cells [46]. The fact that telomeres were not further elongated in *Pot1a*^*+/ki*^ *p53*^*-/-*^ iPS cells compared to the *Pot1a*^*+/+*^ *p53*^*-/-*^ controls, and that both had the same percentage of “signal-free ends”, further supports that longer telomeres in *Pot1a*^*+/ki*^ *p53*^*-/-*^ MEFs are due to increased telomerase access to the telomeres in these cells prior to induction of pluripotency (**Fig 3A).**

To demonstrate the role of telomerase in POT1a p.R117C-induced telomere lengthening, we generated *Pot1*^*+/ki*^ *Tert*^*-/-*^ MEFs. Analysis of telomere length by telomere Q-FISH and by Telomeric Restriction Fragment Southern analysis (TRF) in these cells, clearly shows that the telomere lengthening of *Pot1*^*+/ki*^ MEFs is dependent on telomerase activity, as it does not occur in the double mutant *Pot1*^*+/ki*^ *Tert*^*-/-*^ cells (**Fig 3D–3F**). We observed a decrease in the percentage of short telomeres (< 20^th^ percentile) as well as an increase in the percentage of long telomeres (> 80^th^ percentile) in *Pot1a*^*+/ki*^ compared to *Pot1a*^*+/+*^ MEFs that disappeared in a telomerase deficient background (**Fig 3E**).

Finally, to study a potential role of telomere recombination in the telomere elongation observed in *Pot1a*^*+/ki*^ cells, we next analyzed whether POT1a p.R117C mutant variant affected the recombination rates at telomeres. To this end, we measured the frequency of telomere sister chromatid exchanges (T-SCEs) in MEFs by using the chromosome-orientation FISH (CO-FISH) technique [47]. We found a mild but significant increased numbers of T-SCEs events in *Pot1a*^*+/ki*^ *p53*^*-/-*^ MEFs compared to *Pot1b*^*+/+*^ *p53*^*-/-*^ controls (**Fig 3G**). The presence of C-circles, partially double-stranded telomeric circles consisting of a complete C-rich strand and an incomplete G-rich strand, has also been associated to increased telomere recombination [48]. Thus, we next analyzed the presence of C-circles in *Pot1a*^*+/ki*^*p53*^*-/-*^ and *Pot1b*^*+/+*^*p53*^*-/-*^ MEFs (**S6C Fig**). As positive and negative controls, we used the osteosarcoma U20S cell line and HEK293T that use telomere recombination-based and telomerase-dependent mechanisms for telomere lengthening, respectively [49]. We found increased C-circles in *Pot1a*^*+/ki*^ MEFs that was further enhanced in the absence of p53 although much lower than in ALT+ U2OS cells (**S6C Fig**) [50]. This difference disappeared in *Pot1a*^*+/ki*^ *Tert*^*-/-*^ MEFs (**S6C Fig**). these results indicate that POT1a p.R117C mutant protein is not inducing a *bona fide* ALT mechanism in MEFs.

We also studied the colocalization of PML nuclear bodies with telomeres (ALT-associated PML nuclear bodies or APBs), a phenomenon previously described in cells with increased telomere recombination [7]. We observed higher numbers of APBs in *Pot1a*^*+/ki*^ *p53*^*-/-*^ MEFs as compared to *Pot1a*^*+/+*^ *p53*^*-/-*^, a difference that was not detected in IPS cells, in agreement with telomerasedependent telomere lengthening upon induction of pluripotency (**S6D Fig**). However, the *bona fide* ALT+ U2OS presented a much higher numbers of APBs as compared to *Pot1a*^*+/ki*^ *p53*^*-/-*^, ruling out that expression of POT1a p.R117C mutant protein is favoring the onset of the ALT-mechanism in MEFs.

### Dominant-negative effects of POT1a.R117C mutant protein

**Fig 4:**
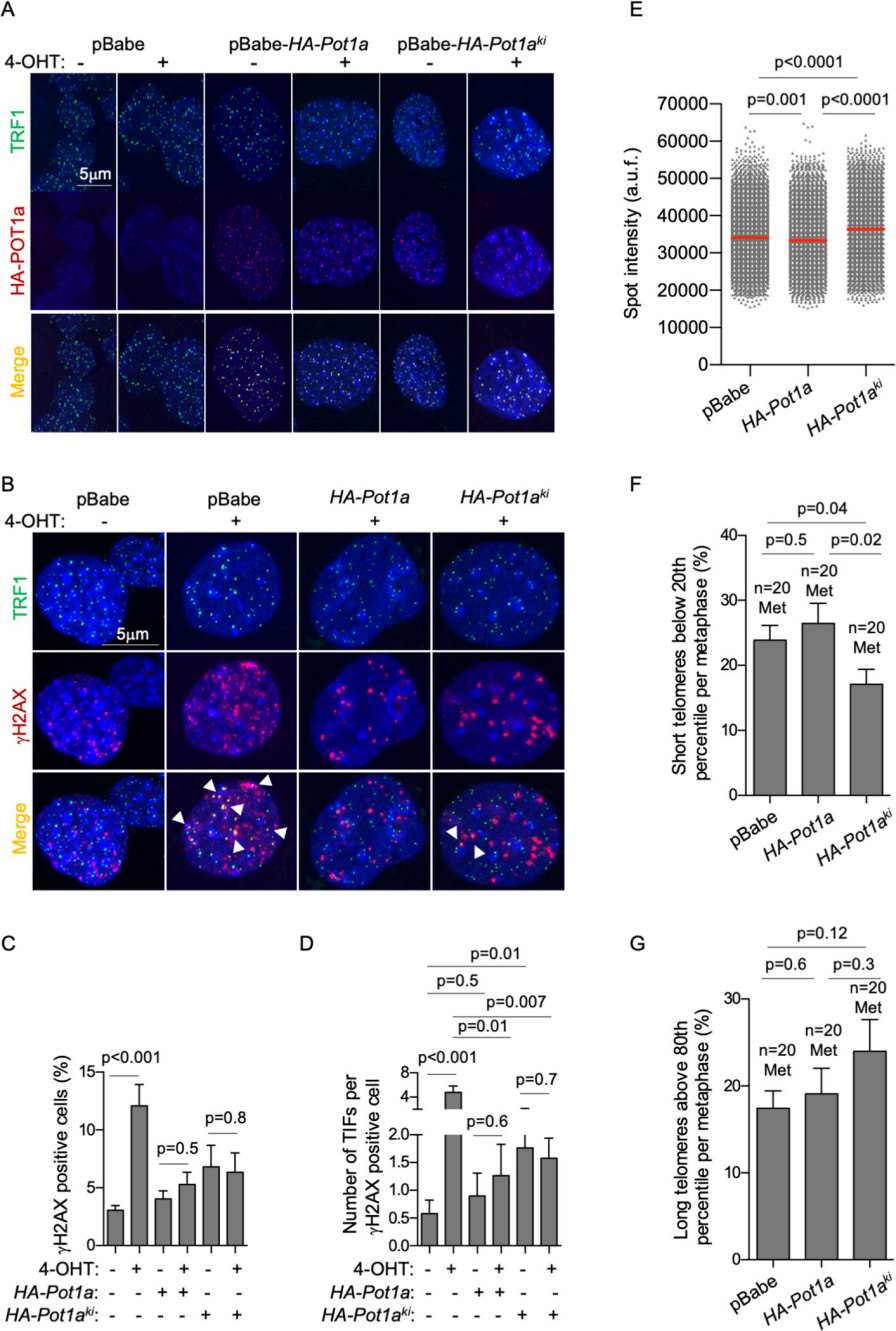
POT1a p.R117C mutant protein exerts dominant-negative effects. **A.** *Pot1a*^*flox/flox*^ MEFs were retrovirally transduced with either empty pBabe, pBabe*-HA-Pot1a* or pBabe-*HA-Pot1a*^*ki*^. After puromycin selection, cells were cultured for 5 days with or without 4-hydroxy-tamoxifen (TMX) to induce the Cre-mediated excision of the floxed *Pot1a* allele. Representative images of double immunofluorescence with antibodies against TRF1 and HA. **B.** Representative images of γH2AX and TRF1 immunofluorescence staining. TIFs were detected by γH2AX and TRF1 co-localizing foci (white arrowheads). **C.** Percentage of γH2AX positive cells. **D.** Number of Telomere-Induced Foci (TIFs) per γH2AX positive cells. **E-G.** Mean spot intensity (E) percentage of short (F) and long (G) telomeres in wild type MEFs overexpressing either *HA-Pot1a* or *HA-Pot1a*^*ki*^ alleles. The percentage of short and long telomeres was defined as fluorescence intensity below the 20^th^ and above 80^th^ percentile, respectively of the fluorescence intensity values of cells transduced with the pBabe empty vector. n = number of metaphases. A t-test two tailed was used for statistical analysis. Error bars represent standard error. The p-values are indicated.

In order to address the nature of the POT1a.pR117C mutant variant we retrovirally transduced *Pot1a*^*flox/flox*^ MEFs with either empty pBabe, pBabe harboring HA-tagged wild-type *Pot1a* or pBabe harboring HA-tagged mutant *Pot1a*^*R117C*^ alleles, pBabe*-HA-Pot1a* or pBabe-*HA-Pot1a*^*ki*^, respectively. After puromycin selection, cells were cultured for 5 days with or without 4-hydroxy-tamoxifen (TMX) to induced the Cre-mediated excision of the floxed flanked exon 4 to exon 5 fragment of the *Pot1a*^*flox*^ allele [37]. We performed a double immunofluorescence using antibodies against TRF1 and HA to address whether the overexpressed *HA-Pot1a* alleles localized to telomeres. The results showed proper co-localization of both HA-POT1a and HA-POT1-KI proteins with TRF1 in both conditions with and without TMX (**Fig 4A**). These results confirm that the R117C mutation does not affect the capacity of POT1-KI mutant protein to localize to telomeres independently of the presence or absence of POT1 wild-type protein.

We next analyzed global genomic DNA damage and telomeric DNA damage (TIFs), which were detected by immunocolocalization of γH2AX and the TRF1 telomere-binding protein (**Fig 4B–4D)**. Cells deleted for *Pot1a* showed a significant increase in global DNA damage and in the number of TIFs. Overexpression of wild-type *HA-Pot1a* and *HA-Pot1a*^*ki*^ alleles rescue both global and telomeric DNA damage, indicating that the R117C substitution does not result in a complete loss of function mutation. Of note, the overexpression of HA-POT1a-KI mutant protein led to a significant 2-fold increase in the number of damaged telomeres as compared to basal levels in pBabe transduced control wild-type cells, a fenomenon that was not detected upon overexpression of wild-type HA-POT1a (**Fig 4D**). These results indicate a dominant-negative effect of the mutant protein as it was also observed in the presence of the endogenous POT1a.

We next set to address the telomere length in wild-type MEFs overexpressing *HA-Pot1a* and *HA-Pot1a*^*ki*^ alleles by Q-FISH on metaphase spreads (**Fig 4E–4G**). Interestingly, overexpression of *HA-Pot1a*^*ki*^ allele led to a significant mean telomere length increase and reduction in the percentage of short telomeres (< 20^th^ percentile) compared to control cells and to cells overexpressing the wild type allele (**Fig 4E and 4F**). In contrast, overexpression of wild type *HA-Pot1a* did not lead to significant changes in the percentage of short telomeres (**Fig 4F**). No significant differences in the percentage of long telomeres (> 80^th^ percentile) were detected among the three experimental conditions (**Fig 4G**). These results reinforce the previous results showing elongation of short telomeres by telomerase as the consequence of the *Pot1a*^*ki*^ expression and indicate that expression of POT1a-R117C mutant variant leads to telomere lengthening.

### *Pot1-ki* mice show longer telomeres

We generated different mouse cohorts carrying the *Pot1a*^*ki*^ mutant allele in heterozygosis both in a p53-proficient and in a p53-deficient background, a situation that is analogous to the human patients carrying this mutation. In particular, we generated mouse cohorts for the *Pot1a*^*+/+*^*p53*^*+/+*^, *Pot1a*^*+/+*^*p53*^*+/-*^, *Pot1a*^*+/+*^*p53*^*-/-*^, *Pot1a*^*+/ki*^*p53*^*+/+*^, *Pot1a*^*+/ki*^*p53*^*+/-*^ and *Pot1a*^*+/ki*^*p53*^*-/-*^ genotypes. Importantly, we found no significant differences in either median or maximal survival between the *Pot1a*^*+/ki*^ cohorts compared to their *Pot1a*^*+/+*^ counterpart controls either in the presence or in the absence of p53 (**S7A Fig**).

**Fig 5:**
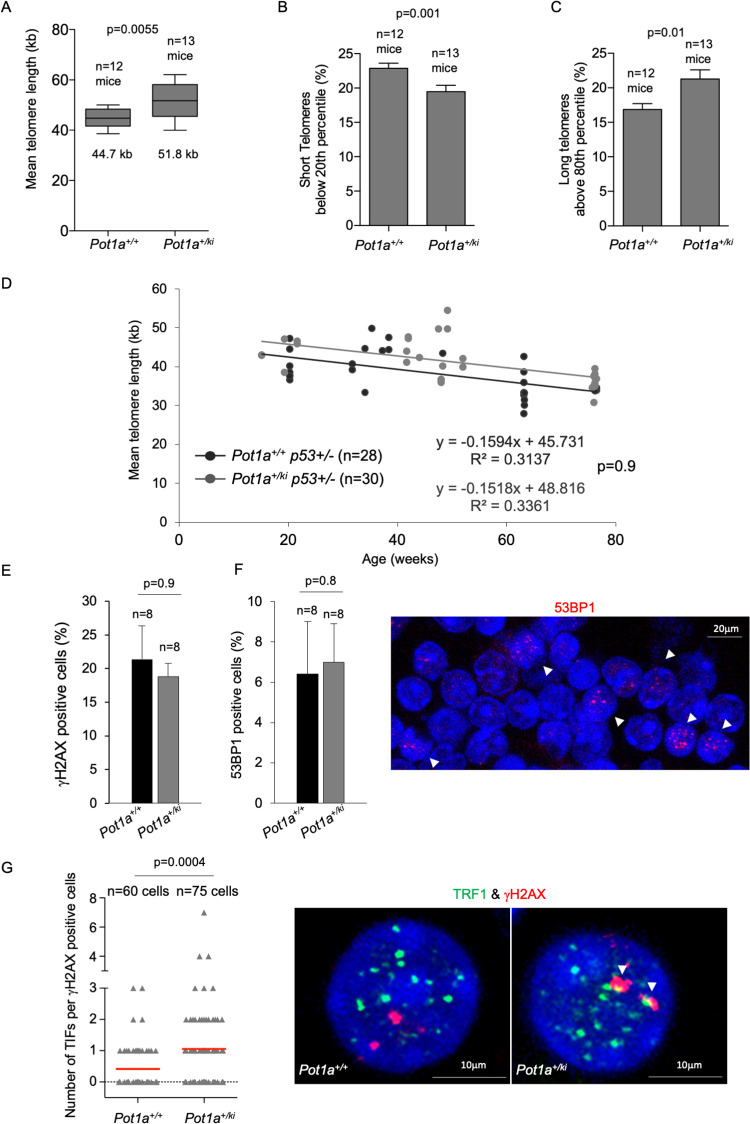
*Pot1a*^*+/ki*^ peripheral blood mononuclear cells show longer telomeres. **A-C.** Whisker plot representation of telomere length (A), percentage of short telomeres (B) and percentage of long telomeres (C) in PMBCs from two-year-old mice of the indicated genotype. The percentage of short and long telomeres was defined as fluorescence intensity below the 20^th^ and above 80^th^ percentile of the fluorescence intensity values of *Pot1a*^*+/+*^ cells, respectively. The ends of the box are the upper and lower quartiles so that the box spans the interquartile range. The middle line represents the median and bars the standard deviation. n = number of mice. **D.** Mean telomere length in PMBCs from mice at different ages from the indicated genotype. Mouse ages range from 15- to 76-week-old. Linear regression analysis was used to determine the rate of telomere shortening. n = number of mice. R^2^ coefficient and the p value from the comparison of both linear regression analysis are indicated. **E-F.** Percentage of γH2AX (E) and 53BP1 (F) positive cells in peripheral blood mononuclear cells (PMBC). n = number of mice. A representative image of 53BP1 staining (white arrowheads) is shown to the right. **G.** Number of Telomere-Induced Foci (TIF) per damaged cells of the indicated genotype. n = number of analyzed cells. Representative images of γH2AX and TRF1 immunofluorescence staining in cells from the indicated genotype are shown to the right. TIFs were detected by γH2AX and TRF1 co-localizing foci (white arrowheads). A t-test two tailed was used for statistical analysis. Error bars represent standard error. The p-values are indicated.

Next, we set to address whether mice carrying the *Pot1a*^*ki*^ allele showed similar telomeric phenotypes to those shown by Li-Fraumeni-Like Syndrome patients carrying the homologous mutation in humans. In particular, we have previously shown that Li-Fraumeni-Like patients show increased telomere length and telomere fragility in peripheral blood mononuclear cells (PBMCs) [13]. Thus, we isolated PBMCs from healthy *Pot1a*^*+/+*^ and *Pot1a*^*+/ki*^ mice at 2 years of age and measured telomere length by using High throughput Telomere Q-FISH (HT-QFISH) which measures telomere fluorescence on interphase nuclei [51]. We found that mean telomere fluorescence was significantly increased in *Pot1a*^*+/ki*^ PBMCs compared to those of control *Pot1a*^*+/+*^ mice, corresponding to a mean length of 51.8 kb compared to 44.7 kb, respectively (**Fig 5A)**. In agreement with longer telomeres in PBMCs from *Pot1a*^*+/ki*^ mice, we observed a significant decrease in the percentage of short telomeres (< 20^th^ percentile) as well as a significant increase in the percentage of long telomeres (> 80^th^ percentile) in *Pot1a*^*+/ki*^ PBMCs compared to those from *Pot1a*^*+/+*^ mice (**Fig 5B and 5C)**. Again, the significant decrease in short telomeres in the PBMCs from *Pot1a*^*+/ki*^ mice, suggest a telomerase-dependent telomere elongation in this genotype.

To further confirm longer telomeres in *Pot1a*^*+/ki*^ mice compared to those of control *Pot1a*^*+/+*^ mice, we determined telomere length in PMBCs from *Pot1a*^*+/+*^*p53*^*+/-*^ and *Pot1a*^*+/ki*^*p53*^*+/-*^ mice at different ages by using HT-QFISH. Again, we observed longer telomeres in *Pot1a*^*+/ki*^ mice compared with *Pot1a*^*+/+*^ mice at all ages analyzed. As expected, the rate of telomere shortening was not significantly different between both mouse cohorts (**Fig 5D**).

As previously shown in the case of MEFs, PBMCs derived from *Pot1a*^*+/ki*^ mice showed a 2-fold increase in telomeric damage (TIFs) as determined by immunocolocalization of γH2AX and the telomeric protein TRF1 compared to the wild-type controls, while the total levels of DNA damage did not vary between genotypes (**Fig 5E–5G**).

**Fig 6:**
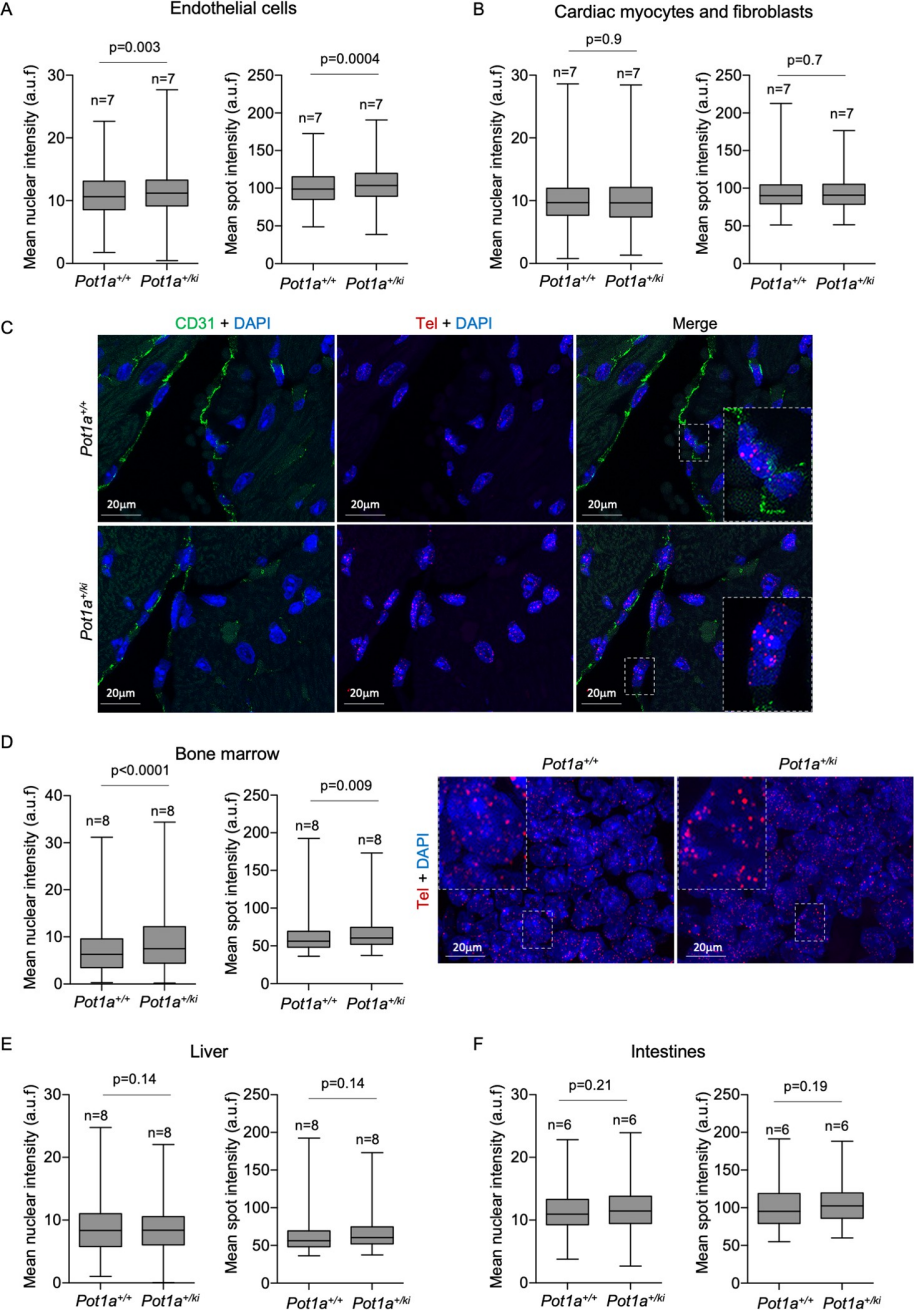
Longer telomeres in the vascular endothelium and in bone marrow cells of *Pot1a*^*+/ki*^ mice. **A-B.** Mean nuclear and mean spot intensity in endothelial cells (A) and in cardiac myocytes and fibroblast (B) from healthy 30-week-old mice of the indicated genotype. **C.** Representative images of immune-FISH from heart sections. The heart sections were stained with anti-CD31 for identification of vascular endothelial cells. **D-F.** Mean nuclear and mean spot intensity in bone marrow (D), liver (E) and intestine (F) from healthy 30-week-old mice of the indicated genotype. Representative images of Q-FISH of bone marrow sections are shown in D. Insets correspond to higher magnification images. n = number of mice. A t-test two tailed was used for statistical analysis. The p-values are indicated.

As human Li-Fraumeni-Like patients show a higher incidence of cardiac angiosarcomas [13], which are originated from endothelial cells, we next analyzed telomere length by Q-FISH in the vascular endothelium. To identify endothelial cells (EC) we used the anti-CD31 marker in heart sections from healthy 30-week-old *Pot1a*^*+/+*^ and *Pot1a*^*+/ki*^ mice. We observed a significantly higher mean nuclear telomere fluorescence and mean telomere spot fluorescence in the vascular endothelium of *Pot1a*^*+/ki*^ compared to wild-type mice indicative of longer telomeres, while no differences in telomere length were detected in CD31 negative cells composed mainly of cardiomyocytes and cardiac fibroblasts [52] (**Fig 6A–6C**).

Finally, we also analyzed telomere length in bone marrow, liver and intestine from the same mouse cohorts. We found longer telomeres in the bone marrow of *Pot1a*^*+/ki*^ mice compared to that of *Pot1a*^*+/+*^ mice while no significant differences in telomere length between both genotypes were observed in liver and intestine (**Fig 6D–6F**).

### Higher incidence of angiosarcomas in *Pot1a*^*+/ki*^ mice

**Fig 7:**
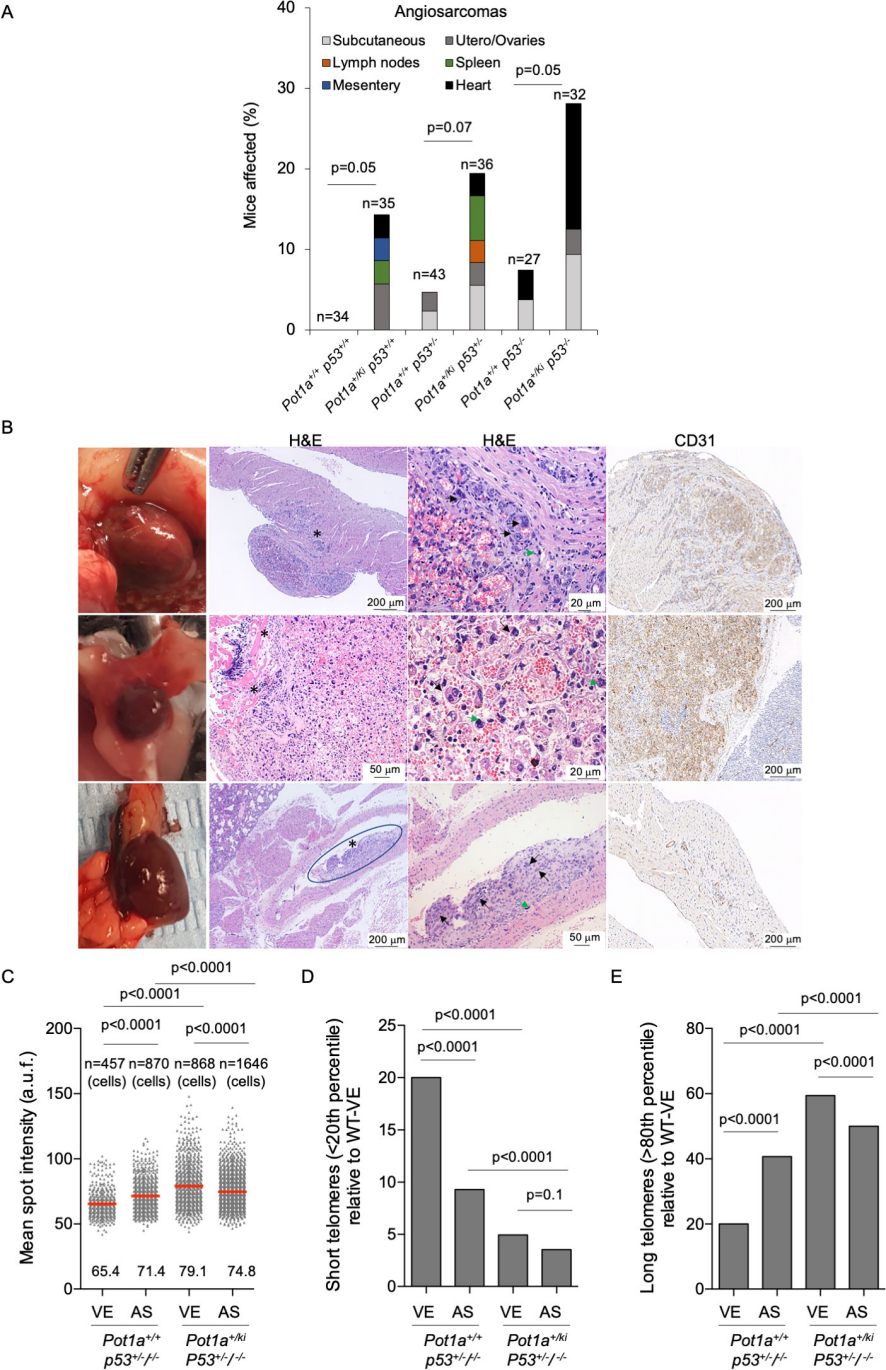
Higher incidence of angiosarcomas in *Pot1a*^*+/ki*^ mice. **A.** Percentage of mice presenting angiosarcomas at time of death. Chi squared test was performed for statistical analysis. The number of mice and the p values are indicated. **B.** Macroscopic and representative images of hematoxilin & eosin (H&E) and CD31 staining of angiosarcomas developed in vascular endothelium within the thorax cavity. Dark-red angiosarcoma protrudes from right ventricular wall (upper panels), on the pleural surface of the sternum (middle panels) and in the pulmonary artery (lower panels). Malignant endothelial cells have invaded surrounding tissues (asterisk). High magnification images show anaplastic endothelial cells with large and hyperchromatic nuclei (black arrows) forming capillary and sinusoidal channels. Mitotic figures were observed (green arrows). CD31 was used as a vascular marker for diagnosis. Scale bars are shown. **C-D.** Mean telomere fluorescence intensity (C), percentage of short telomeres (D) and percentage of long telomeres (E) in healthy vascular endothelium (VE) and in angiosarcomas (AS) from mice of the indicated genotype at death point. The percentage of short and long telomeres was defined as fluorescence intensity below the 20^th^ and above 80^th^ percentile, respectively of the fluorescence intensity values of the wild type VE. n = number of cells. Two *Pot1a*^*+/+*^ tumors were analyzed, a utero angiosarcoma (*Pot1a*^*+/+*^*p53*^*+/-*^) and a cardiac angiosarcoma (*Pot1a*^*+/+*^*p53*^*-/-*^). Four *Pot1a*^*+/ki*^ thoracic angiosarcomas were analyzed, three *Pot1a*^*+/ki*^*p53*^*-/-*^ and one *Pot1a*^*+/ki*^*p53*^*+/-*^. Non-tumoral vascular endothelium from the same mice were also analyzed. A t-test two tailed was used for statistical analysis in C and a Fisher’s exact test for the percentage of short and long telomeres. The p-values are indicated.

As the human *POT1*^*R117C*^ heterozygous mutation is associated with higher incidence of cancers including familial cardiac angiosarcomas [13], here we set to address whether the equivalent mouse *Pot1a*^*R117C*^ mutation also led to increased tumors in the context of mice carrying the *Pot1a*^*ki*^ allele in heterozygosis. To this end, we performed a full histopathological analysis of mice of the different genotypes at death point and analyzed the frequency of spontaneous tumors. Mice carrying the *Pot1a*^*ki*^ allele showed an increased angiosarcoma incidence within the thorax cavity, including heart angiosarcomas (**Fig 7A and 7B**). Of note, while none of the *Pot1a*^*+/+*^ mice in a p53 wild-type background developed angiosarcoma, a total of 14.3% of the *Pot1a*^*+/ki*^ mice developed these tumors (**Fig 7A)**. This higher incidence of angiosarcomas in *Pot1a*^*+/ki*^ mice compared to wild-type mice was also observed in the p53 heterozygous and homozygous genetic backgrounds (**Fig 7A)**. We used a positive CD31 staining, a marker for vascular endothelial cells, for diagnosis of angiosarcoma incidence (**Fig 7B**). The thoracic angiosarcomas developed by *Pot1a*^*+/ki*^ mice were characterized by invasion of malignant endothelial cells to surrounding tissues and the presence of anaplastic endothelial cells with large and hyperchromatic nuclei forming capillary and sinusoidal channels (see “black arrow” in **Fig 7B**). These tumors also showed a number of mitotic figures (see “green arrow” in **Fig 7B**). Of note, the incidence of thoracic angiosarcomas is low and the size of these tumors are very small, making it impossible to check for angiosarcoma at earlier time points. It is of interest to note the fact that the increased angiosarcoma incidence is in accordance with increased telomere length in endothelial cells expressing the *Pot1a*^*R117C*^ mutation, and indicate that mice carrying this mutation are a *bona fide* model to study the pathobiology of these tumors, as well as potential effective treatments.

Mice carrying the *Pot1a*^*R117C*^ mutation also presented rare carcinomas in mice such as lung bronchoalveolar carcinoma and hypophysis carcinomas, although the frequency was not significantly different between genotypes (**S7B Fig**). Also, the incidence of benign, sarcoma and hematopoietic tumors was not significantly different when comparing *Pot1a*^*+/+*^ with *Pot1a*^*+/ki*^ mouse cohorts in none of the p53 background (**S7C–S7E Fig**).

### Longer telomeres in *Pot1-ki* angiosarcomas

As endothelial cells from the heart of *Pot1a*^*+/ki*^ mice showed longer telomeres, we next set to determine telomere length in angiosarcomas from both *Pot1a*^*+/+*^ and *Pot1a*^*+/ki*^ mice at death point. Angiosarcoma tumors expressing POT1a p.R117C showed significantly longer telomeres compared to similar tumors found in wild-type mice (**Fig 7C**). Non-tumoral vascular endothelium surrounding the tumors were also analyzed and we found that *Pot1a*^*+/ki*^ endothelial cells have longer telomeres compared to wild-type endothelial cells (**Fig 7C**; see also **Fig 6A**). Accordingly, the percentage of short telomeres (< 20^th^ percentile) was lower in *Pot1a*^*ki*^ angiosarcomas (AS) and healthy vascular epithelium (VE), while the percentage of long telomeres (> 80^th^ percentile) was higher in *Pot1a*^*ki*^ AS and in VE as compared to those in wild-type mice (**Fig 7D and 7E**). The longer telomeres in *Pot1a*^*ki*^ AS are consistent with the telomerase-mediated telomere elongation found in both MEFs and iPS cells carrying the POT1a p.R117C mutation (**Fig 3A–3F**).

Interestingly, we did not find longer telomeres in hypophysis and lung adenomas and carcinomas compared to their surrounding healthy tissue from *Pot1a*^*+/ki*^ mice (**S8A–S8C Fig**). Of note, telomeres where shorter in tumor samples compared to their corresponding non-pathological tissue (**S8A–S8C Fig**), which is consistent with previous studies [53]. We did not detect PML positive cells within the carcinomas (**S8D Fig**).

As found in *Pot1a*^*+/ki*^ MEFs, we also observed increased PML-positive cells and higher number of APBs in *Pot1a*^*+/ki*^ VE and AS compared to *Pot1a*^*+/+*^ control samples, respectively (**S9A–S9C Fig**). Of note, the number of APB foci per cell increased significantly in *Pot1a*^*+/ki*^ angiosarcomas compared to non-pathological vascular epithelium (**S9A–S9C Fig**).

Finally, we also analyzed total DNA damage and telomeric damage by γH2AX and TRF1 double immunofluorescence in angiosarcoma tumors. Overall DNA damaged was low, 5% γH2AX positive cells of total tumoral cells. Of note, no differences either in the percentage of damaged cells or in Telomere-Induced DNA damage foci (TIFs) between *Pot1a* wild-type and *knock-in* angiosarcomas were detected (**S9D and S9E Fig**), indicating that telomeric DNA damage in *Pot1a*^*ki*^ angiosarcomas is not contributing to tumorigenesis.

## Discussion

The availability of mouse models that recapitulate the human disease is instrumental to understand the origin and molecular mechanisms associated to the development of a particular disease, as well as to develop effective treatments. In this work we have generated a mouse model for humans carrying the *hPOT1*^*R117C*^ mutation associated to Li-Fraumeni like syndrome by generating a *Pot1a*^*R117C*^ *knock-in* mouse. Among other tumor types, families carrying the *hPOT1*^*R117C*^ mutation show an elevated incidence of cardiac angiosarcomas, a rare tumor type [13]. Although additional POT1 variants have been found in cardiac angiosarcomas and cardiac sarcomas, *in silico* studies predict that these variants are affecting the POT1 function in a similar way than the p.R117C mutation studied here [12]. In all cases, these mutations acted as dominant-negative alleles, as indicated by absence of loss of heterozygosity in the corresponding tumors [12,13]. Thus, here we generated a heterozygous *Pot1a*^+/*ki*^ mouse model and showed that mouse POT1a pR117C exerts dominant-negative effects at telomeres. Importantly, we show that these mice spontaneously develop angiosarcomas, including cardiac angiosarcoma, but not other tumor types, at a higher frequency than wild-type mice. These observations indicate that the homologous missense mutation R117C found in *hPOT1* introduced in *mPot1a* has similar consequences in angiosarcoma development in human and mouse.

It is of particular relevance that *POT1* mutations are mainly associated to increased angiosarcomas both in mice and humans. In this regard, a recent pan-cancer study aimed at to identify associations between *POT1* mutation frequency and tumor type, found that 1834 tumors out of a total of 62,368 tumors harbored a non-benign mutation in POT1 (2.94%) of which angiosarcomas turned out to be 11 times more likely to carry a *POT1* mutation than other cancer types [22]. The highest frequency of *POT1* mutation was found in pulmonary sarcomatoid carcinomas (28%) and angiosarcomas (23%) [21], which are particularly aggressive malignancies compared with other types of sarcomas in which the frequency of *POT1* mutations was not elevated. Thus, pathological POT1 mutant variants seem to be associated to the increased aggressiveness of pulmonary sarcomatoid carcinomas and angiosarcomas [21]. In an independent study, the Angiosarcoma Project (ASCproject) in which altered genes in a cohort of 47 angiosarcomas were studied, POT1 mutations were the fourth most frequent after mutations in *TP53*, *KDR* and *PIK3CA* genes [54].

We and others have shown that at the molecular level, the *hPOT1* mutations found in human tumors cause telomere dysfunction, genomic instability and telomere lengthening [13,16,18,20,23,27,28], which might facilitate survival of cells with chromosomal instability and the acquisition of aditional somatic mutations leading to malignant transformation [29]. Here, we demonstrated that *Pot1a*^*R117C*^ mice carrying the p.R117C mutation in heterozygosis recapitulate the longer telomere phenotype found in humans. In particular, we observed longer telomeres in lymphocytes, as well as in mouse embryonic fibroblasts (MEFs), cardiac endothelial cells and bone marrow cells. We did not find longer telomeres, however, in liver, intestines and cardiac muscle. It would be of interest to know whether human carriers of these mutation show increased telomere length in other tissues other than lymphocytes.

Longer telomeres as the consequence of the *Pot1a*^*R117C*^ mutation could be the consequence of increased recruitment or accessibility of telomerase to the telomeres. Human POT1 functions both as a positive and a negative regulator of telomerase since POT1 and its interacting partner TPP1 have been shown to have a role in recruiting telomerase to telomeres as well as to inhibit telomerase access to telomeres [33,35,55–57]. Alternatively, *POT1* mutations may also affect telomere length throughout its role in recruitment of the CST complex that binds to telomeric DNA and regulates the fill-in synthesis of the telomeric C-strand [28,30,58]. In mice, POT1b interacts with TPP1 to promote telomerase recruitment to telomeres to elongate telomeric G-strand as well as C-strand fill-in through CST complex recruitment while POT1a negatively regulates telomerase access to telomeres [59]. The fact that telomerase deficient MEFs carrying the *Pot1a*^*R117C*^ mutation do not present longer telomeres compared to *Pot1a* wild-type counterparts demonstrate that telomere lengthening induced by POT1a pR117C is telomerase dependent. Given that the *ki-Pot1a*^*R117C*^ mouse conserves intact POT1b, the longer telomeres observed are likely due to a defect of POT1a p.R117C in preventing of telomerase access to telomeres and/or an enhanced function as a telomerase activator/processivity factor bound to TPP1. Also, the reduced abundance of undetectable telomere signals at chromosome ends (“signal-free ends”) in *Pot1a*^*+/ki*^ compared with wild-type MEFs suggests a higher telomerase activity at telomeres as it has previously been shown that telomerase preferentially acts on the shortest telomeres both in mice and yeast [44–46]. In agreement with this, the difference in the percentage of “signal-free ends” between wild-type and *Pot1a*^*R117C*^ genotypes is lost upon induction of pluripotency, which involves an opening of the telomeric chromatin and a net telomerase-mediated telomere elongation [46].

Interestingly, we also find longer telomeres in *Pot1a*^*R117C*^ angiosarcomas, which are also likely the consequence of telomerase-elongation associated to the *POT1a*^*R117C*^ mutation as shown here for *Pot1a*^*R117C*^ MEFs. Of interest, although telomerase over-expression if the most widely used telomere elongating mechanism in tumors, some cancers of mesenchymal and neuroepithelial cell origin [60], such as angiosarcomas [61,62], have been reported to show an Alternative Lengthening of Telomeres (ALT) phenotype. Interestingly, here we find higher numbers of APBs in angiosarcomas forming in *Pot1a*^*R117C*^ mice, suggesting that increased recombination could also be partially contributing to telomere elongation in these tumors. We cannot rule out that the cancer phenotype associated to *Pot1*^*R117C*^ in the mouse model could also be due to telomere dysfunction resulting from suboptimal end protection by the POT1a pR117C variant that could cause telomere recombination and other forms of genome instability that are causative of cancer. Other cancer associated POT1 mutations when introduced in stem cells do not cause significant telomere damage but led to telomere elongation [63]. Based on the fact that the *Pot1a*^*+/R117C*^ mice don not present an increase incidence in either carcinomas, sarcomas or hematological tumors, and only a significant increase in angiosarcomas in adulthood, we propose that POT1a p.R117C variant is not the driver but rather a facilitator of neoplastic transformation in endothelial cells. Cancer-associated POT1 mutations by their ability to lengthen telomeres might endow cancer cells with proliferative advantages.

Finally, it is interesting to note that although different hPOT1 variants studied lead to similar telomeric phenotypes, i.e. longer telomeres and telomere fragility [13,16,18,20,23,27,28], the families carrying a particular *POT1* mutation do not show a wide spectrum of POT1-associated tumors as it could be expected [11–20,22,24–26,64]. This is also the case of the *Pot1a*^+/*R117C*^ mice generated here, which spontaneously develop angiosarcomas but the incidence of other type of sarcomas, carcinomas or hematological tumors was not altered compared to wild-type mice.

In summary, the *ki-Pot1a* ^*R117C*^ mouse constitutes a potential pre-clinical mouse model for LFL syndrome presenting with high angiosarcoma incidence that could provide in the future a very useful tool for the study of treatments for these tumors.

## Materials and methods

### Ethics statement

All mice were generated and maintained at the Spanish National Cancer Centre under specific pathogen-free conditions in accordance with the recommendation of the Federation of European Laboratory Animal Science Associations. All experiments and animal procedures were approved by our Institutional Animal Care and Use Committee (IACUC) and by the Ethics Committee for Research and Animal Welfare (CEIyBA) (PROEX 065/16).

### Generation of *Pot1a* ^*R117C*^ knock-in mouse model

The *knock-in* for *Pot1a*^*FRT-Neo/flexR117C*^ was generated following a conditional strategy by the Flex technology [65]. The targeting construct was produced by Gene Bridges (www.genebridges.com). G4-ES cells were electroporated with 10 μg of *Asc*I linearized targeting vector. ES clones showing homologous recombination at the *Pot1a* locus was identified by PCR. The results of PCR screening were confirmed by Southern blot analysis of EcoR I restricted genomic DNA using a 5’- and a 3’- external probes, respectively (**S1A and S1B Fig**). Unique insertion of the targeting vector was confirmed using a probe within the Neomycin marker (**S1C Fig**). The integrity of the distal loxP511 site was confirmed by PCR amplification of genomic DNA from the selected ES clones using primers Pot1a-1Fand Pot1a-R1 (**S1 Table**) and sequencing the PCR products (**S1D Fig**). Chimeric mice were generated by microinjection of two independently targeted ES clones into B6N-Tyr^C-Brd^ host blastocyst, which were then implanted into pseudopregnant CD1 foster females. The resulting offspring showed a high level of chimerism as shown by coat color, and were mated to C57BL/6J mice to asses germ line transmission. The resulting heterozygous *Pot1a*^*+/FRT-Neo*.*flexR117C*^ mice were then bred to transgenic mice expressing the Flpe recombinase to induce excision of the Neo marker to generate *Pot1a*^*+/*.*flexR117C*^ mice (**S1E Fig**). *Pot1a*^*+/*.*flexR117C*^ heterozygous mice were then intercrossed to generate *Pot1a*^*flexR117C/*.*flexR117C*^ resulting in embryonic lethality. *Pot1a*^*+/*.*flexR117C*^ heterozygous mice were crossed with transgenic mice expressing the Cre recombinase under the control of the adenovirus EIIa promoter [38] to generate *Pot1a*^*+/*.*ki-R117C*^, hereafter named *Pot1a*^*+/ki*^. *Pot1a*^*+*^ and *Pota*^*ki*^ alleles were genotyped by PCR analysis using primers F2 and R1 (**S1 Table**). Amplification of the wild type and knock-in alleles renders a 892 bp and 963 bp fragments, respectively. The F2-R1 Ki fragment contains a PstI restriction site that is absent in wild type allele and that upon cleavage results in two fragments of 399 bp and 565 bp respectively (**S1F Fig**). The *Pot1a*^*+/ki*^ heterozygous was back-crossed with pure C57BL/6J background mice to achieve 96,88% C57BL/6J background. Two independent *Pot1a*^*ki*^ mouse colonies originated from two selected recombinant stem cell clones were generated as described above. No phenotypic differences were observed between both colonies and therefore the results throughout this work correspond to pooled data from both colonies. The *Pot1a*^*+/ki*^ were crossed with *p53*^*-/-*^ and to *Tert*^*-/-*^ to generate the double compound *Pot1a*^*+/ki*^ *p53*^*-/-*^ and *Pot1a*^*+/ki*^ *Tert*^*-/-*^ mice, respectively [66].

### Cell culture and Induced Pluripotent stem (iPs) cell generation

Primary embryonic fibroblasts (MEFs) were isolated from E11.5 or E13.5 embryos according to standard protocols. Briefly, after removal of the head and organs the whole embryo was minced and rinsed in ice-cold PBS, incubated in trypsin/EDTA (Gibco, Grand Island, NY) before dissociating in complete medium. MEFs were grown in high-glucose DMEM supplemented with 10% FBS. For iPS cell generation, MEFs were transduced with retroviral supernatants produced in 293T cells transfected with the ecotropic packaging plasmid pCL-Eco and the Yamanaka factors, OCT4, SOX2 or KLF4 [40]. *Pot1a*^*flox/flox*^ MEFs were transduced with retroviral supernatants produced in 293T cells transfected with the ecotropic packaging plasmid pCL-Eco and with either empty pBabe, pBabe*-HA-Pot1a-6xHIS* or pBabe-*HA-Pot1aki-6xHIS*. Cells were cultured for 5 days with or without 4-hydroxy-tamoxifen (1 μM). The *HA-Pot1a-6xHIS* and *HA-Pot1a*^*ki*^*-6xHIS* alleles were synthetically synthesized by Integrated DNA Technologies, IDT (https://eu.idtdna.com) and cloned in pBabe vector.

### Immunohistochemistry and Immunofluorescence analysis

Tissue samples were fixed in 10% buffered formalin, dehydrated, embedded in paraffin wax and sectioned at 2.5 mm. Tissue sections were deparaffinized in xylene and re-hydrated through a series of graded ethanol until water and then stained with hematoxylin and eosin for pathological examination. Immunohistochemistry (IHC) and immunofluorescence (IF) were performed on de-paraffined tissue sections processed with 10 mM sodium citrate (pH 6.5) cooked under pressure for 2 min. IHC staining of angiosarcoma sections was performed with rabbit polyclonal anti-CD31 (1:100; Abcam ab28354), counterstained with hematoxylin and analyzed by light microscopy. For IF, Tissue sections were permeabilized with 0.5% Triton in PBS and blocked with 5% BSA in PBS. Lymphocytes, MEFs and IPs cells were plated in Poly-L-lysine-coated coverslips, treated for 5 min with Triton-100 buffer for nuclear extraction, fixed 10 min in 4% buffered formaldehyde, permeabilized with 0.2% PBS-Triton for 10 min and blocked with 5% BSA in PBS for 1h. Samples were incubated O/N at 4°C with rabbit polyclonal anti-TRF1 (1:500; CNIO homemade), with rabbit polyclonal anti-CD31 (1:50; Abcam ab28354), mouse monoclonal anti-phospho-histone γH2A.X-Ser139 (1:300) (Millipore, 05–636), rabbit-polyclonal anti-53BP1 (1:300; Novus Biologicals, NB100-304) or with rabbit polyclonal anti-PML (1:100; Santa Cruz Biotechnology, H-238). When indicated, a Q-Fish was performed on IF stained slides fixed with 4% formaldehyde for 20 minutes.

MEFs and IPS cells were treated for 5 min with Triton-100 buffer for nuclear extraction, fixed 10 min in 4% buffered formaldehyde, permeabilized with 0.2% PBS-Triton for 10 min and blocked with 5% fetal bovine serum in PBS for 1h. Samples were incubated O/N at 4°C with rabbit anti-TRF1 (1:500) (CNIO homemade antibody), mouse anti-γH2A.X-Ser139 (1:500) (05–636, Millipore), with rabbit anti-PML (1:100) (Santa Cruz Biotechnology, H-238) and mouse anti-HA-Tag (1:100) (Cell Signalling, 2367). Cells were then washed and incubated with 488-Alexa or 555-Alexa labeled secondary antibodies (Thermo Fisher Scientific) for 1 h at RT in a humid chamber. Samples were mounted in Prolong Gold with DAPI (Invitrogen).

Immunofluorescence images were obtained using a confocal laser-scanning microscope (Leica TSC SP5) using a Plan Apo 63Å-1.40 NA oil immersion objective (HCX). Maximal projection of z-stack images generated using advanced fluorescence software (LAS) were analyzed with Definiens XD software package.

### Quantitative Fluorescence In Situ Hybridization (Q-FISH) analysis

For quantitative telomere fluorescence *in situ* hybridization (Q-FISH), paraffin-embedded tissue sections were deparaffinized and fixed with 4% formaldehyde, followed by digestion with pepsin/HCl and a second fixation with 4% formaldehyde. Slides were dehydrated with increasing concentrations of EtOH (70%, 90%, 100%) and incubated with the telomeric (TTAGGG) probe labelled with Cy3 at 85°C for 3 min followed by 2h at room temperature in a wet chamber. The slides were extensively washed with 50% formamide and 0.08% TBS-Tween 20. Confocal microscopy was performed at room temperature with a laser-scanning microscope (Leica TSC SP5) using a Plan Apo 63Å-1.40 NA oil immersion objective (HCX). Maximal projection of z-stack images generated using advanced fluorescence software (LAS) were analyzed with Definiens XD software package. The DAPI images were used to detect telomeric signals inside each nucleus.

High-throughput (HT)-QFISH on peripheral blood leukocytes was done using 150 μl of blood as described [51]. Confocal images were captured using the Opera High-Content Screening system (Perkin Elmer).

MEFs and iPS cells were incubated with 0.1 μg/ml colcemide during 4 h and 2h, respectively. After hypotonic swelling in 0.03 M sodium citrate for 30 min at 37°C, cells were fixed in methanol:acetic acid (3:1). Quantitative telomere fluorescence in situ hybridization (Q-FISH) was performed as described. Images were captured using microscope Leica DM6B using a 100x oil objective. Telomere length was analyzed using Leica Application Suite X Software. The incidence of chromosomal aberrations per metaphase was determined by eye. The images were analyzed blindly.

### Telomere recombination measurements using chromosome orientation FISH (CO-FISH)

Exponentially growing primary MEFs were sub-cultured in the presence of 5’-bromo-2’-deoxyuridine (BrdU; Sigma) at a final concentration of 1x10^-5^ M, and then allowed to replicate their DNA once at 37°C for 12 hours. Colcemide was added at a concentration of 0.1 μg/ml during the last 4 hours. Cells were then recovered and metaphases prepared as described [44].

### Western blot analysis

Total cell and nuclear protein extracts were obtained by RIPA extraction buffer MERK, R0278) or using a Nuclear/Cytosolic Fractionation Kit (Biovision, K266-100) and protein concentration was determined using a Bradford Reagent (B6916, Sigma Aldrich). Forty micrograms of nuclear extracts were separated in 4–12% SDS-PAGE gels (NuPAGE Invitrogen) and transferred to nitrocellulose membranes (Amersham Protan). Blots were incubated with the indicated antibodies. Antibody binding was detected after incubation with a secondary antibody coupled to horseradish peroxidase using a chemiluminescence with ECL detection kit (GE Healthcare). The primary antibodies used were rat monoclonal anti-POT1a (1:200, Clone name POP148C, CNIO homemade), rat monoclonal anti-TRF1 (1:500, Clone name 572C, CNIO homemade), rabbit polyclonal anti-nanog (1:1000, Cell Signaling, 8822), rabbit polyclonal antiphospho-CHK1 (Ser345) (1:1000, Cell Signaling, 2348), mouse monoclonal anti-CHK1 (1:1000, Cell Signaling, 2360), rabbit polyclonal anti-phosphoRPA32 (1:1000, Bethyl), A300-245A), rat monoclonal anti-RPA32 (1:1000, Cell Signalling, 2208) and rabbit polyclonal anti-SMC1 (1:8000; Bethyl, A300-055A).

### RNA and qPCR

Total RNA from cells was extracted with the RNeasy kit (74106, QIAGEN) and reverse transcribed was using the iSCRIPT cDNA synthesis kit (1708891, BIO-RAD) according to manufacturer’s protocol. Quantitative real-time PCR was performed with the QuantStudio 6 Flex (Applied Biosystens, Life Technologies) using Go-Taq Green Master Mix (M7123, Promega) according to the manufacturer’s protocol. All values were obtained in triplicates. Primer sequences can be found in **S1 Table**. We determined the relative expression in each sample by calculating the 2ΔCT value. For each sample, 2ΔCT was normalized to control 2ΔCT mean.

### Chromatin Immune Precipitation assay (ChIP)

Formaldehyde was added directly to MEFs culture medium to a final concentration of 1% and incubated for 15 min at room temperature (RT) on a shaking platform. Cross-linking was then stopped by addition of glycine to a final concentration of 0.125 M for 5 min at RT. Cross-linking cells were washed twice with cold PBS containing 1 μM PMSF and protease inhibitors and then pelleted. Cells were lysed in lysis buffer (1% SDS, 10 mM EDTA and 50 mM Tris-HCl pH 8.0) containing protease inhibitors for 20 min at 4°C. Lysates were sonicated to obtain chromatin fragments <1 kb and centrifuged for 15 min in a microfuge at room temperature. Chromatin was diluted 1:10 with dilution buffer (1.1% Triton X-100, 2 mM EDTA pH 8.0, 150 mM NaCl, 20 mM Tris-HCl pH 8.0) and precleared with 50 μl of protein A/G Plus-Agarose beads (sc-2003, Santa Cruz Biotechnology). After centrifugation, chromatin fragments were incubated at 4°C overnight on a rotating platform with rat monoclonal anti-POT1a (CNIO homemade), rabbit polyclonal anti-TRF1 (CNIO homemade), rabbit polyclonal anti-TRF2 (1:1000, Novus Biologicals, NB110-57130,) or with rabbit IgG (sc-2025, Santa Cruz Biotechnology). Samples were then immunoprecipitated with 50 μl of protein A/G Plus-Agarose beads. The immunoprecipitated pellets were washed once with IP Wash A (0.1% SDS, 1% Triton X-100, 2 mM EDTA, 20 mM Tris-HCl pH 8.0), IP Wash B (150 mM NaCl, and then with 0.1% SDS, 1% Triton X-100, 2 mM EDTA, 20 mM Tris-HCl pH 8.0), IP Wash C (500 mM NaCl, 0.25 M LiCl, 1% Nonidet P-40, 1% sodium deoxycholate, 1 mM EDTA, and 10 mM Tris-HCl pH 8.0) and finally with TE (10 mM Tris-HCl pH 8.0 and 1 mM EDTA) two times. The chromatin was eluted from the beads twice by incubation with 250 μl 1% SDS and 0.05 M NaHCO_3_ during 15 min at RT with rotation. After adding 20 μl of 5 M NaCl, the crosslink was reversed overnight at 65°C. Samples were supplemented with 20 μl of 1 M Tris-HCl pH 6.5, 10 μl of 0.5 M EDTA, 20 μg of RNase A, and 40 μg of proteinase K, and incubated for 1 h at 45°C. DNA was recovered by phenol–chloroform extraction and ethanol precipitation, denatured (0.3 M NaOH) at 50°C for 1 h, neutralized (1 M ammonium acetate), and transferred to a Hybond-N+ membrane (Amersham) on a dot blot. The membranes were hybridized with a telomeric probe obtained from a plasmid containing 1.6 kb of TTAGGG repeats (gift from T. de Lange, Rockefeller University). The signal was quantified with the ImageJ software. The amount of telomeric DNA immunoprecipitated was calculated in each ChIP based on the signal relative to the corresponding total telomeric DNA signal.

### C-circle assay

One μg of genomic DNA was digested with HinfI and RsaI in the presence of RNase. Genomic DNA (200 ng) was diluted in phi29 reaction buffer (0.2 mg/ ml BSA, 0.1% Tween, 1mM each dATP, dGTP and dTTP, 1xphi29 buffer) (total volume of 40 μl) and 10 units of 4BB QualiPhi DNA polymerase (4basebio) were added. The amplification reaction was performed at 30°C for 8 hours and inactivated at 65°C for 20 min. The reaction product was diluted with 2xSSC to 120 μl and dot-blotted on an N-Hybond (Amersham). DNA was UV-cross-linked onto the membrane which was hybridized at 57°C with ^32^P-(CCCTAA)_7_ in Church and Gilbert hybridization buffer (1% BSA, 1mM EDTA, 7% SDS, 15% formamide and 200 mM sodium phosphate pH 7.2).

## Supporting information

S1 Fig *Pot1a knock-in* mouse generation. Genomic DNA was digested with EcoR1 restriction enzymes for southern analysis and the size of the fragments are indicated. **A-C.** Southern blot images from 5’- (A), 3’- (B) and neo (C) specific probes from two independent targeted stem cell clones (clone 47 and clone 52). **D.** Agarose gel of PCR product using primers F1 and R1 encompassing the distal LoxP511 site from the selected stem cell clones. These PCR products were further sequenced to assure the 3’-end of the targeted allele was not lost in the genome integration event. **E.** Southern blot image with the 3’-probe from EcoRI digested genomic DNA from mice after excision of the Neo cassette by the FLP recombinase. These mice were used as the parental mice for generating the *Pot1a*^*+/flex*^ colonies. **F.** Schematic representation of the PCR genotyping reaction for the *Pot1a*^*+*^ and *Pot1a*^*ki*^ alleles. A representative image of an agarose gel showing the PstI cleaved PCR amplification products using primers upstream and downstream E7 (F2 and R1, respectively) from genomic DNA from *Pot1a*^*+/+*^ and *Pot1a*^*+/ki*^ mice after cross with the EIIa-Cre tool mouse.
(TIFF)

S2 Fig Expression levels of *Pot1a* alleles. **A.** Expected and observed number of mice of the offspring from *Pot1a*^*+/flex*^ intercrosses. **B-C.** Expected and observed number of embryos of the offspring from *Pot1a*^*+/flex*^ (B) and from *Pot1a*^*+/ki*^*p53*^*-/-*^ (C) intercrosses. The Fisher’s exact test was used to determine statistical significance. p-values are indicated**. D.** Schematic representation of *Pot1a*, *Pot1a*^*ki*^ and *Pot1a*^*flex*^ mRNA. The R117C substitution is within exon 7 (E7*). Five different primers pairs were used to quantify transcripts levels corresponding to E3-E5 (green arrows), E7 (black and red arrows for E7 wildtype and E7*, respectively), E8-E9 (purple arrows) and E9-E10 (brown arrows). The scheme is not drawn to scale. **E.** Quantification of expression levels by qRT-PCR of different exons in *Pot1a*, *Pot1a*^*ki*^ and *Pot1a*^*flex*^ alleles in tail tissue of *Pot1a*^*+/+*^, *Pot1a*^*+/ki*^ and *Pot1a*^*+/flex*^ mice. A t-test two tailed was used for statistical analysis. The p-value is indicated. Mean values +/- SEM are represented. N = number of mice analyzed per genotype.
(TIFF)

S3 Fig The *Pot1a*^*ki*^ allele is properly transcribed. **A.** Nucleotide BLAST alignment of *Pot1a* wild type and mutant knock-in exon 7 sequences. The mutant exon 7 contains several silent substitutions and one missense mutation that results in an amino acid change from arginine to cysteine at position 117. **B**. Sequencing spectrum of wild type (upper panel) and mutant (lower panel) *Pot1a*-exon 7. The cDNA from *Pot1a*^*+/+*^ and *Pot1a*^*+/ki*^ MEFs was PCR amplified using primers annealing within exon 3 (Pot1a E3-E5-F) and within exon9 (Pot1a E8-E9-R) (S1 Table). The PCR products were subjected to Sanger sequencing. The 5’ and 3’ ends of exon 7 and are indicated.
(TIFF)

S4 Fig POT1a mutant protein binds telomeric DNA *in vivo*. **A-C.** Quantification of telomeric DNA pulled down with anti-TRF1(A), with anti-TRF2 (B) and with anti-POT1a (C) of MEFs of the indicated genotype. DNA input signal is also shown. **D.** Representative images of chromatin immunoprecipitation (ChIP) of telomeric DNA. Low and high exposure images are shown. ChIP values are normalized by the input of each individual sample. **E-F.** Quantification of TRF1 fluorescence intensity levels in intestines (E) and in liver (F) of mice of the indicated genotype. Representative images of TRF1 immunofluorescence are shown below. Bars and error bars represent mean values ± SE. N  =  number of independent experiments. Student’s t-test was used for the statistical analysis. P-values are indicated.
(TIFF)

S5 Fig Generation of induced pluripotent stem (iPS) cells. **A.** Quantification and representative western blot images of nuclear extracts of TRF1 and Nanog protein levels in *Pot1a*^*+/+*^*p53*^*-/-*^ and *Pot1a*^*+/ki*^*p53*^*-/-*^ MEFs and iPS cells. SMC1 was used as loading control. IPS cells were generated from two independent MEFs from each genotype. The protein level quantification is represented in the bar plot. **B**. Quantification of *Tert* expression levels by q-RT-PCR in MEFs and IPs of the indicated genotype. Two MEFs and two iPS cells from each genotype were used for the analysis. The analysis was performed four times for each cell line. n = replicates. Two tailed Student’s t-test was used for the statistical analysis. P-values are indicated.
(TIFF)

S6 Fig POT1a p.R117C expression does not lead to ATR activation. **A,B**. Representative western blot images of total cellular extracts of phosphor-CHK1, total CHK1, phospo-RPA, total RPA protein levels in *Pot1a*^*+/+*^*p53*^*-/-*^ and *Pot1a*^*+/ki*^*p53*^*-/-*^ MEFs (A) and IPSs (B). SMC1 was used as loading control. Cells were treated with hydroxyurea (2 mM) for 3 hours as positive controls for replicative stress. **C.** C-circle quantification and representative dot-blot images of MEFs of the indicated genotype. U2OS and HEK293T cells were used as positive and negative controls, respectively. A negative control without Phi29 polymerase is also shown. The C-circle score is calculated as the percentage of the signal relative to that of ALT positive U2OS cell line. n = number of independent experiments **D.** Number of ALT-associated PML bodies (APBs) in PML positive cells (E) in MEFs of the indicated genotype. n = number of cells. Representative Immune-Fish images of PML and a telomeric probe. APBs were detected by PML and Telomere co-localizing foci (white arrowheads). A t-test two tailed was used for statistical analysis. Error bars represent standard error. The p-value is indicated.
(TIFF)

S7 Fig Pot1a *knock-in* mice do not present higher incidence of benign, hematopoietic and sarcoma tumors. **A.** Kaplan-Meier survival curves of mouse cohorts of the indicated genotypes. Median survival values are indicated. A Log-rank (Mantel-Cox) test was performed for statistical analysis. P- values are indicated. n = number of mice. **B.** Percentage of mice presenting carcinomas at death. Representative H&E images of a hypophysis carcinoma are shown to the right. The neoplasm invades hypothalamus and the thalamus (asterisk). Hemorrhagic areas were present (red arrow). Atypical polygonal cells arranged in solid sheets with fibrovascular stroma. The tumor cells showed pleomorphic nuclei, large nucleoli (black arrow) and high number of mitosis (green arrows) were observed. Chi squared test was performed for statistical analysis. The number of mice and the p values are indicated. Scale bars are shown. **C-E.** Percentage of mice of the indicated genotype presenting benign (C), hematopoietic (D) and sarcoma (E) tumors at death. The types of tumors are indicated in the legend. Chi squared test was performed for statistical analysis. None of the comparisons were statistically significant (ns). N = number of mice.
(TIFF)

S8 Fig Shorter telomeres in tumors than in surrounding healthy tissue. **A.** Telomere mean spot intensity in hypophysis tumors and in surrounding non-tumoral hypophysis tissue. Three adenomas from *Pot1a*^*+/ki*^*p53*^*+/+*^ and one carcinoma from *Pot1a*^*+/ki*^*p53*^*-/-*^ mice at death were analyzed. **B.** Telomere mean spot intensity in lung tumors and in surrounding non-tumoral lung tissue. One lung adenoma and two bronchoalveolar carcinomas from *Pot1a*^*+/ki*^*p53*^*+/+*^ mice at death were analyzed. **C.** Telomere mean spot intensity in stomach carcinomas and in surrounding non-tumoral stomach tissue. Two *Pot1a*^*+/ki*^*/p53*^*+/-*^ stomach carcinomas were analyzed. The box-and-whisker graph shows the values lower and greater than first and 99^th^ percentile for each group. Mean spot intensity is indicated in each case. n = number of cells. A t-test two tailed was used for statistical analysis. The p-values are indicated. **D.** Representative Immune-Fish images of PML and a telomeric probe in carcinomas from hypophysis, lung and stomach. Bone marrow sections were used as staining positive control. Insets correspond to higher magnification images.
(TIFF)

S9 Fig Higher incidence of ALT-associated PML bodies in *Pot1a*^*+/ki*^ angiosarcomas. **A-B.** Percentage of PML positive cells (A) and number of ALT-associated PML bodies (APBs) per cell (B) in healthy vascular endothelium (VE) and in angiosarcomas (AS) from mice of the indicated genotype at death point. A PML positive cell was defined as having >2 foci. Two *Pot1a*^*+/+*^ tumors were analyzed, a utero angiosarcoma (*Pot1a*^*+/+*^*p53*^*+/-*^) and a cardiac angiosarcoma (*Pot1a*^*+/+*^*p53*^*-/-*^). Four *Pot1a*^*+/ki*^ thoracic angiosarcomas were analyzed, three *Pot1a*^*+/ki*^*p53*^*-/-*^ and one *Pot1a*^*+/ki*^*p53*^*+/-*^. Non-tumoral vascular endothelium from the same mice were also analyzed. **F.** Representative Immune-Fish images of PML and a telomeric probe in vascular endothelium and in cardiac angiosarcomas of the indicated genotypes. Vascular endothelium was identified by autofluorescence of red blood cells within the blood vessels. APBs were detected by PML and Telomere co-localizing foci (white arrowheads). **D-E.** Percentage of γH2AX positive cells (D) and number of Telomere-Induced Foci (TIF) per cell (E) in AS from mice of the indicated genotype at death point. The samples analyzed were the same as in A. A representative image of γH2AX and TRF1 staining is shown. TIFs were detected by γH2AX and TRF1 co-localizing foci. A t-test two tailed was used for statistical analysis. The p-values are indicated.
(TIFF)

S1 Table Primers used in this study. (DOCX)

S1 Data Numerical data underlying graphs. (XLSX)

## References

[1] Blasco MA. The epigenetic regulation of mammalian telomeres. Nat Rev Genet. 2007;8(4):299–309. doi: 10.1038/nrg2047 . 17363977

[2] de Lange T. Shelterin: the protein complex that shapes and safeguards human telomeres. Genes Dev. 2005;19(18):2100–10. doi: 10.1101/gad.1346005 . 16166375

[3] Martinez P, Blasco MA. Telomeric and extra-telomeric roles for telomerase and the telomere-binding proteins. Nat Rev Cancer. 2011;11(3):161–76. Epub 2011/02/25. nrc3025 [pii] doi: 10.1038/nrc3025 . 21346783

[4] Lopez-Otin C, Blasco MA, Partridge L, Serrano M, Kroemer G. The hallmarks of aging. Cell. 2013;153(6):1194–217. Epub 2013/06/12. S0092-8674(13)00645-4 [pii] doi: 10.1016/j.cell.2013.05.039 ; PubMed Central PMCID: PMC3836174. 23746838 PMC3836174

[5] Blackburn EH. Switching and signaling at the telomere. Cell. 2001;106(6):661–73. doi: 10.1016/s0092-8674(01)00492-5 . 11572773

[6] Hanahan D, Weinberg RA. The hallmarks of cancer. Cell. 2000;100(1):57–70. Epub 2000/01/27. S0092-8674(00)81683-9 [pii]. doi: 10.1016/s0092-8674(00)81683-9 . 10647931

[7] Cesare AJ, Reddel RR. Alternative lengthening of telomeres: models, mechanisms and implications. Nat Rev Genet. 2010;11(5):319–30. Epub 2010/03/31. doi: 10.1038/nrg2763 . 20351727

[8] Barthel FP, Wei W, Tang M, Martinez-Ledesma E, Hu X, Amin SB, et al. Systematic analysis of telomere length and somatic alterations in 31 cancer types. Nat Genet. 2017;49(3):349–57. Epub 2017/01/31. doi: 10.1038/ng.3781 ; PubMed Central PMCID: PMC5571729. 28135248 PMC5571729

[9] Allory Y, Beukers W, Sagrera A, Flandez M, Marques M, Marquez M, et al. Telomerase reverse transcriptase promoter mutations in bladder cancer: high frequency across stages, detection in urine, and lack of association with outcome. Eur Urol. 2014;65(2):360–6. Epub 2013/09/11. S0302-2838(13)00904-4 [pii] doi: 10.1016/j.eururo.2013.08.052 . 24018021

[10] Huang DS, Wang Z, He XJ, Diplas BH, Yang R, Killela PJ, et al. Recurrent TERT promoter mutations identified in a large-scale study of multiple tumour types are associated with increased TERT expression and telomerase activation. Eur J Cancer. 2015;51(8):969–76. Epub 2015/04/07. doi: 10.1016/j.ejca.2015.03.010 [pii] ; PubMed Central PMCID: PMC4467782. 25843513 PMC4467782

[11] Bainbridge MN, Armstrong GN, Gramatges MM, Bertuch AA, Jhangiani SN, Doddapaneni H, et al. Germline mutations in shelterin complex genes are associated with familial glioma. J Natl Cancer Inst. 2014;107(1):384. Epub 2014/12/09. dju384 [pii] doi: 10.1093/jnci/dju384 ; PubMed Central PMCID: PMC4296199. 25482530 PMC4296199

[12] Calvete O, Garcia-Pavia P, Dominguez F, Bougeard G, Kunze K, Braeuninger A, et al. The wide spectrum of POT1 gene variants correlates with multiple cancer types. Eur J Hum Genet. 2017;25(11):1278–81. Epub 2017/08/31. doi: 10.1038/ejhg.2017.134 ; PubMed Central PMCID: PMC5643968. 28853721 PMC5643968

[13] Calvete O, Martinez P, Garcia-Pavia P, Benitez-Buelga C, Paumard-Hernandez B, Fernandez V, et al. A mutation in the POT1 gene is responsible for cardiac angiosarcoma in TP53-negative Li-Fraumeni-like families. Nat Commun. 2015;6:8383. Epub 2015/09/26. ncomms9383 [pii] doi: 10.1038/ncomms9383 ; PubMed Central PMCID: PMC4598567. 26403419 PMC4598567

[14] Chubb D, Broderick P, Dobbins SE, Frampton M, Kinnersley B, Penegar S, et al. Rare disruptive mutations and their contribution to the heritable risk of colorectal cancer. Nat Commun. 2016;7:11883. Epub 2016/06/23. doi: 10.1038/ncomms11883 ; PubMed Central PMCID: PMC4917884. 27329137 PMC4917884

[15] Kataoka K, Nagata Y, Kitanaka A, Shiraishi Y, Shimamura T, Yasunaga J, et al. Integrated molecular analysis of adult T cell leukemia/lymphoma. Nat Genet. 2015;47(11):1304–15. Epub 2015/10/06. doi: 10.1038/ng.3415 . 26437031

[16] McMaster ML, Sun C, Landi MT, Savage SA, Rotunno M, Yang XR, et al. Germline mutations in Protection of Telomeres 1 in two families with Hodgkin lymphoma. Br J Haematol. 2018;181(3):372–7. Epub 2018/04/26. doi: 10.1111/bjh.15203 ; PubMed Central PMCID: PMC5921926. 29693246 PMC5921926

[17] Newey PJ, Nesbit MA, Rimmer AJ, Attar M, Head RT, Christie PT, et al. Whole-exome sequencing studies of nonhereditary (sporadic) parathyroid adenomas. J Clin Endocrinol Metab. 2012;97(10):E1995–2005. Epub 2012/08/03. jc.2012-2303 [pii] doi: 10.1210/jc.2012-2303 ; PubMed Central PMCID: PMC4446457. 22855342 PMC4446457

[18] Ramsay AJ, Quesada V, Foronda M, Conde L, Martinez-Trillos A, Villamor N, et al. POT1 mutations cause telomere dysfunction in chronic lymphocytic leukemia. Nat Genet. 2013;45(5):526–30. Epub 2013/03/19. ng.2584 [pii] doi: 10.1038/ng.2584 . 23502782

[19] Richard MA, Lupo PJ, Morton LM, Yasui YA, Sapkota YA, Arnold MA, et al. Genetic variation in POT1 and risk of thyroid subsequent malignant neoplasm: A report from the Childhood Cancer Survivor Study. PLoS One. 2020;15(2):e0228887. Epub 2020/02/11. doi: 10.1371/journal.pone.0228887 . 32040538 PMC7010302

[20] Robles-Espinoza CD, Harland M, Ramsay AJ, Aoude LG, Quesada V, Ding Z, et al. POT1 loss-of-function variants predispose to familial melanoma. Nat Genet. 2014;46(5):478–81. Epub 2014/04/02. ng.2947 [pii] doi: 10.1038/ng.2947 ; PubMed Central PMCID: PMC4266105. 24686849 PMC4266105

[21] Shen E, Xiu J, Bentley R, Lopez GY, Walsh KM. Frequent Mutations of POT1 Distinguish Pulmonary Sarcomatoid Carcinoma From Other Lung Cancer Histologies. Clin Lung Cancer. 2020;21(6):e523–e7. Epub 2020/05/18. doi: 10.1016/j.cllc.2020.04.002 . 32414627

[22] Shen E, Xiu J, Lopez GY, Bentley R, Jalali A, Heimberger AB, et al. POT1 mutation spectrum in tumour types commonly diagnosed among POT1-associated hereditary cancer syndrome families. J Med Genet. 2020;57(10):664–70. Epub 2020/01/16. doi: 10.1136/jmedgenet-2019-106657 ; PubMed Central PMCID: PMC7427478. 31937561 PMC7427478

[23] Shi J, Yang XR, Ballew B, Rotunno M, Calista D, Fargnoli MC, et al. Rare missense variants in POT1 predispose to familial cutaneous malignant melanoma. Nat Genet. 2014;46(5):482–6. Epub 2014/04/02. ng.2941 [pii] doi: 10.1038/ng.2941 ; PubMed Central PMCID: PMC4056593. 24686846 PMC4056593

[24] Speedy HE, Di Bernardo MC, Sava GP, Dyer MJ, Holroyd A, Wang Y, et al. A genome-wide association study identifies multiple susceptibility loci for chronic lymphocytic leukemia. Nat Genet. 2014;46(1):56–60. Epub 2013/12/03. doi: 10.1038/ng.2843 . 24292274

[25] Wong K, Robles-Espinoza CD, Rodriguez D, Rudat SS, Puig S, Potrony M, et al. Association of the POT1 Germline Missense Variant p.I78T With Familial Melanoma. JAMA Dermatol. 2019;155(5):604–9. Epub 2018/12/27. doi: 10.1001/jamadermatol.2018.3662 ; PubMed Central PMCID: PMC6506889. 30586141 PMC6506889

[26] Zhang J, Jima D, Moffitt AB, Liu Q, Czader M, Hsi ED, et al. The genomic landscape of mantle cell lymphoma is related to the epigenetically determined chromatin state of normal B cells. Blood. 2014;123(19):2988–96. Epub 2014/04/01. doi: 10.1182/blood-2013-07-517177 [pii] ; PubMed Central PMCID: PMC4014841. 24682267 PMC4014841

[27] Gu P, Wang Y, Bisht KK, Wu L, Kukova L, Smith EM, et al. Pot1 OB-fold mutations unleash telomere instability to initiate tumorigenesis. Oncogene. 2017;36(14):1939–51. Epub 2016/11/22. doi: 10.1038/onc.2016.405 ; PubMed Central PMCID: PMC5383532. 27869160 PMC5383532

[28] Pinzaru AM, Hom RA, Beal A, Phillips AF, Ni E, Cardozo T, et al. Telomere Replication Stress Induced by POT1 Inactivation Accelerates Tumorigenesis. Cell Rep. 2016;15(10):2170–84. Epub 2016/05/31. S2211-1247(16)30561-7 [pii] doi: 10.1016/j.celrep.2016.05.008 . 27239034 PMC6145145

[29] Calvete O, Garcia-Pavia P, Dominguez F, Mosteiro L, Perez-Cabornero L, Cantalapiedra D, et al. POT1 and Damage Response Malfunction Trigger Acquisition of Somatic Activating Mutations in the VEGF Pathway in Cardiac Angiosarcomas. J Am Heart Assoc. 2019;8(18):e012875. Epub 2019/09/13. doi: 10.1161/JAHA.119.012875 ; PubMed Central PMCID: PMC6818007. 31510873 PMC6818007

[30] Chen LY, Redon S, Lingner J. The human CST complex is a terminator of telomerase activity. Nature. 2012;488(7412):540–4. Epub 2012/07/06. doi: 10.1038/nature11269 . 22763445

[31] Kelleher C, Kurth I, Lingner J. Human protection of telomeres 1 (POT1) is a negative regulator of telomerase activity in vitro. Mol Cell Biol. 2005;25(2):808–18. Epub 2005/01/06. doi: 10.1128/MCB.25.2.808-818.2005 ; PubMed Central PMCID: PMC543404. 15632080 PMC543404

[32] Lei M, Podell ER, Cech TR. Structure of human POT1 bound to telomeric single-stranded DNA provides a model for chromosome end-protection. Nat Struct Mol Biol. 2004;11(12):1223–9. Epub 2004/11/24. nsmb867 [pii] doi: 10.1038/nsmb867 . 15558049

[33] Tejera A, Stagno d´Alcontres M, Marion RM, Thanasoula M, Martinez P, Liao C, et al. TPP1 is required for TERT recruitment, telomere elongation during nuclear reprogramming, and normal skin development in mice. Developmental Cell. 2010;18(5):691–702. 20493811 10.1016/j.devcel.2010.03.011 PMC3631760

[34] Hockemeyer D, Daniels JP, Takai H, de Lange T. Recent expansion of the telomeric complex in rodents: Two distinct POT1 proteins protect mouse telomeres. Cell. 2006;126(1):63–77. doi: 10.1016/j.cell.2006.04.044 . 16839877

[35] He H, Wang Y, Guo X, Ramchandani S, Ma J, Shen MF, et al. Pot1b deletion and telomerase haploinsufficiency in mice initiate an ATR-dependent DNA damage response and elicit phenotypes resembling dyskeratosis congenita. Mol Cell Biol. 2009;29(1):229–40. doi: 10.1128/MCB.01400-08 . 18936156 PMC2612488

[36] Hockemeyer D, Palm W, Else T, Daniels JP, Takai KK, Ye JZ, et al. Telomere protection by mammalian Pot1 requires interaction with Tpp1. Nat Struct Mol Biol. 2007;14(8):754–61. Epub 2007/07/17. doi: 10.1038/nsmb1270 . 17632522

[37] Wu L, Multani AS, He H, Cosme-Blanco W, Deng Y, Deng JM, et al. Pot1 deficiency initiates DNA damage checkpoint activation and aberrant homologous recombination at telomeres. Cell. 2006;126(1):49–62. doi: 10.1016/j.cell.2006.05.037 . 16839876

[38] Lakso M, Pichel JG, Gorman JR, Sauer B, Okamoto Y, Lee E, et al. Efficient in vivo manipulation of mouse genomic sequences at the zygote stage. Proc Natl Acad Sci U S A. 1996;93(12):5860–5. Epub 1996/06/11. doi: 10.1073/pnas.93.12.5860 ; PubMed Central PMCID: PMC39152. 8650183 PMC39152

[39] Martinez P, Thanasoula M, Munoz P, Liao C, Tejera A, McNees C, et al. Increased telomere fragility and fusions resulting from TRF1 deficiency lead to degenerative pathologies and increased cancer in mice. Genes Dev. 2009;23(17):2060–75. Epub 2009/08/15. doi: 10.1101/gad.543509 [pii] ; PubMed Central PMCID: PMC2751970. 19679647 PMC2751970

[40] Takahashi K, Yamanaka S. Induction of pluripotent stem cells from mouse embryonic and adult fibroblast cultures by defined factors. Cell. 2006;126(4):663–76. Epub 2006/08/15. doi: 10.1016/j.cell.2006.07.024 . 16904174

[41] Schneider RP, Garrobo I, Foronda M, Palacios JA, Marion RM, Flores I, et al. TRF1 is a stem cell marker and is essential for the generation of induced pluripotent stem cells. Nat Commun. 2012;4:1946. Epub 2013/06/06. ncomms2946 [pii] doi: 10.1038/ncomms2946 . 23735977

[42] Munoz P, Blanco R, Flores JM, Blasco MA. XPF nuclease-dependent telomere loss and increased DNA damage in mice overexpressing TRF2 result in premature aging and cancer. Nat Genet. 2005;37(10):1063–71. Epub 2005/09/06. doi: 10.1038/ng1633 . 16142233

[43] Sfeir A, Kosiyatrakul ST, Hockemeyer D, MacRae SL, Karlseder J, Schildkraut CL, et al. Mammalian telomeres resemble fragile sites and require TRF1 for efficient replication. Cell. 2009;138(1):90–103. Epub 2009/07/15. doi: 10.1016/j.cell.2009.06.021 ; PubMed Central PMCID: PMC2723738. 19596237 PMC2723738

[44] Samper E, Flores JM, Blasco MA. Restoration of telomerase activity rescues chromosomal instability and premature aging in Terc-/- mice with short telomeres. EMBO Rep. 2001;2(9):800–7. doi: 10.1093/embo-reports/kve174 ; PubMed Central PMCID: PMC1084029. 11520856 PMC1084029

[45] Teixeira MT, Arneric M, Sperisen P, Lingner J. Telomere length homeostasis is achieved via a switch between telomerase- extendible and -nonextendible states. Cell. 2004;117(3):323–35. Epub 2004/04/28. doi: 10.1016/s0092-8674(04)00334-4 . 15109493

[46] Marion RM, Strati K, Li H, Tejera A, Schoeftner S, Ortega S, et al. Telomeres acquire embryonic stem cell characteristics in induced pluripotent stem cells. Cell Stem Cell. 2009;4(2):141–54. Epub 2009/02/10. S1934-5909(09)00002-2 [pii] doi: 10.1016/j.stem.2008.12.010 . 19200803

[47] Bailey SM, Goodwin EH, Cornforth MN. Strand-specific fluorescence in situ hybridization: the CO-FISH family. Cytogenet Genome Res. 2004;107(1–2):14–7. Epub 2004/08/12. doi: 10.1159/000079565 . 15305050

[48] Henson JD, Cao Y, Huschtscha LI, Chang AC, Au AY, Pickett HA, et al. DNA C-circles are specific and quantifiable markers of alternative-lengthening-of-telomeres activity. Nat Biotechnol. 2009;27(12):1181–5. Epub 2009/11/26. doi: 10.1038/nbt.1587 . 19935656

[49] Henson JD, Reddel RR. Assaying and investigating Alternative Lengthening of Telomeres activity in human cells and cancers. FEBS Lett. 2010;584(17):3800–11. Epub 2010/06/15. doi: 10.1016/j.febslet.2010.06.009 . 20542034

[50] Jiang WQ, Zhong ZH, Nguyen A, Henson JD, Toouli CD, Braithwaite AW, et al. Induction of alternative lengthening of telomeres-associated PML bodies by p53/p21 requires HP1 proteins. J Cell Biol. 2009;185(5):797–810. Epub 2009/05/27. doi: 10.1083/jcb.200810084 ; PubMed Central PMCID: PMC2711592. 19468068 PMC2711592

[51] Canela A, Vera E, Klatt P, Blasco MA. High-throughput telomere length quantification by FISH and its application to human population studies. Proc Natl Acad Sci U S A. 2007;104(13):5300–5. Epub 2007/03/21. doi: 10.1073/pnas.0609367104 ; PubMed Central PMCID: PMC1828130. 17369361 PMC1828130

[52] Zhou P, Pu WT. Recounting Cardiac Cellular Composition. Circ Res. 2016;118(3):368–70. Epub 2016/02/06. doi: 10.1161/CIRCRESAHA.116.308139 ; PubMed Central PMCID: PMC4755297. 26846633 PMC4755297

[53] de Lange T, Shiue L, Myers RM, Cox DR, Naylor SL, Killery AM, et al. Structure and variability of human chromosome ends. Mol Cell Biol. 1990;10(2):518–27. Epub 1990/02/01. doi: 10.1128/mcb.10.2.518-527.1990 PubMed Central PMCID: PMC360828. 2300052 PMC360828

[54] Painter CA, Jain E, Tomson BN, Dunphy M, Stoddard RE, Thomas BS, et al. The Angiosarcoma Project: enabling genomic and clinical discoveries in a rare cancer through patient-partnered research. Nat Med. 2020;26(2):181–7. Epub 2020/02/12. doi: 10.1038/s41591-019-0749-z . 32042194

[55] Wang F, Podell ER, Zaug AJ, Yang Y, Baciu P, Cech TR, et al. The POT1-TPP1 telomere complex is a telomerase processivity factor. Nature. 2007;445(7127):506–10. Epub 2007/01/24. doi: 10.1038/nature05454 . 17237768

[56] Loayza D, De Lange T. POT1 as a terminal transducer of TRF1 telomere length control. Nature. 2003;423(6943):1013–8. doi: 10.1038/nature01688 . 12768206

[57] Nandakumar J, Cech TR. Finding the end: recruitment of telomerase to telomeres. Nat Rev Mol Cell Biol. 2013;14(2):69–82. Epub 2013/01/10. nrm3505 [pii] doi: 10.1038/nrm3505 ; PubMed Central PMCID: PMC3805138. 23299958 PMC3805138

[58] Pinzaru AM, Kareh M, Lamm N, Lazzerini-Denchi E, Cesare AJ, Sfeir A. Replication stress conferred by POT1 dysfunction promotes telomere relocalization to the nuclear pore. Genes Dev. 2020;34(23–24):1619–36. Epub 2020/10/31. doi: 10.1101/gad.337287.120 ; PubMed Central PMCID: PMC7706707. 33122293 PMC7706707

[59] Gu P, Jia S, Takasugi T, Tesmer VM, Nandakumar J, Chen Y, et al. Distinct functions of POT1 proteins contribute to the regulation of telomerase recruitment to telomeres. Nat Commun. 2021;12(1):5514. Epub 2021/09/19. doi: 10.1038/s41467-021-25799-7 ; PubMed Central PMCID: PMC8448735. 34535663 PMC8448735

[60] Heaphy CM, Subhawong AP, Hong SM, Goggins MG, Montgomery EA, Gabrielson E, et al. Prevalence of the alternative lengthening of telomeres telomere maintenance mechanism in human cancer subtypes. Am J Pathol. 2011;179(4):1608–15. Epub 2011/09/06. doi: 10.1016/j.ajpath.2011.06.018 ; PubMed Central PMCID: PMC3181356. 21888887 PMC3181356

[61] Heaphy CM, de Wilde RF, Jiao Y, Klein AP, Edil BH, Shi C, et al. Altered telomeres in tumors with ATRX and DAXX mutations. Science. 2011;333(6041):425. Epub 2011/07/02. doi: 10.1126/science.1207313 ; PubMed Central PMCID: PMC3174141. 21719641 PMC3174141

[62] Liau JY, Tsai JH, Yang CY, Lee JC, Liang CW, Hsu HH, et al. Alternative lengthening of telomeres phenotype in malignant vascular tumors is highly associated with loss of ATRX expression and is frequently observed in hepatic angiosarcomas. Hum Pathol. 2015;46(9):1360–6. Epub 2015/07/21. doi: 10.1016/j.humpath.2015.05.019 . 26190196

[63] Kim WT, Hennick K, Johnson J, Finnerty B, Choo S, Short SB, et al. Cancer-associated POT1 mutations lead to telomere elongation without induction of a DNA damage response. The EMBO journal. 2021;40(12):e107346. Epub 2021/05/03. doi: 10.15252/embj.2020107346 ; PubMed Central PMCID: PMC8204863. 33934394 PMC8204863

[64] Shih IM, Zhou W, Goodman SN, Lengauer C, Kinzler KW, Vogelstein B. Evidence that genetic instability occurs at an early stage of colorectal tumorigenesis. Cancer Res. 2001;61(3):818–22. Epub 2001/02/28. . 11221861

[65] Schnutgen F, Doerflinger N, Calleja C, Wendling O, Chambon P, Ghyselinck NB. A directional strategy for monitoring Cre-mediated recombination at the cellular level in the mouse. Nat Biotechnol. 2003;21(5):562–5. Epub 2003/04/01. doi: 10.1038/nbt811 . 12665802

[66] Liu Y, Snow BE, Hande MP, Yeung D, Erdmann NJ, Wakeham A, et al. The telomerase reverse transcriptase is limiting and necessary for telomerase function in vivo. Curr Biol. 2000;10(22):1459–62. Epub 2000/12/05. doi: 10.1016/s0960-9822(00)00805-8 . 11102810

