# Supplementary figures and images for: A mouse model for Li-Fraumeni-Like Syndrome with cardiac angiosarcomas associated to POT1 mutations
Source: PLoS Genet. 2022 Jun 21;18(6):e1010260. doi: 10.1371/journal.pgen.1010260 (PMC9212151; doi:10.1371/journal.pgen.1010260)

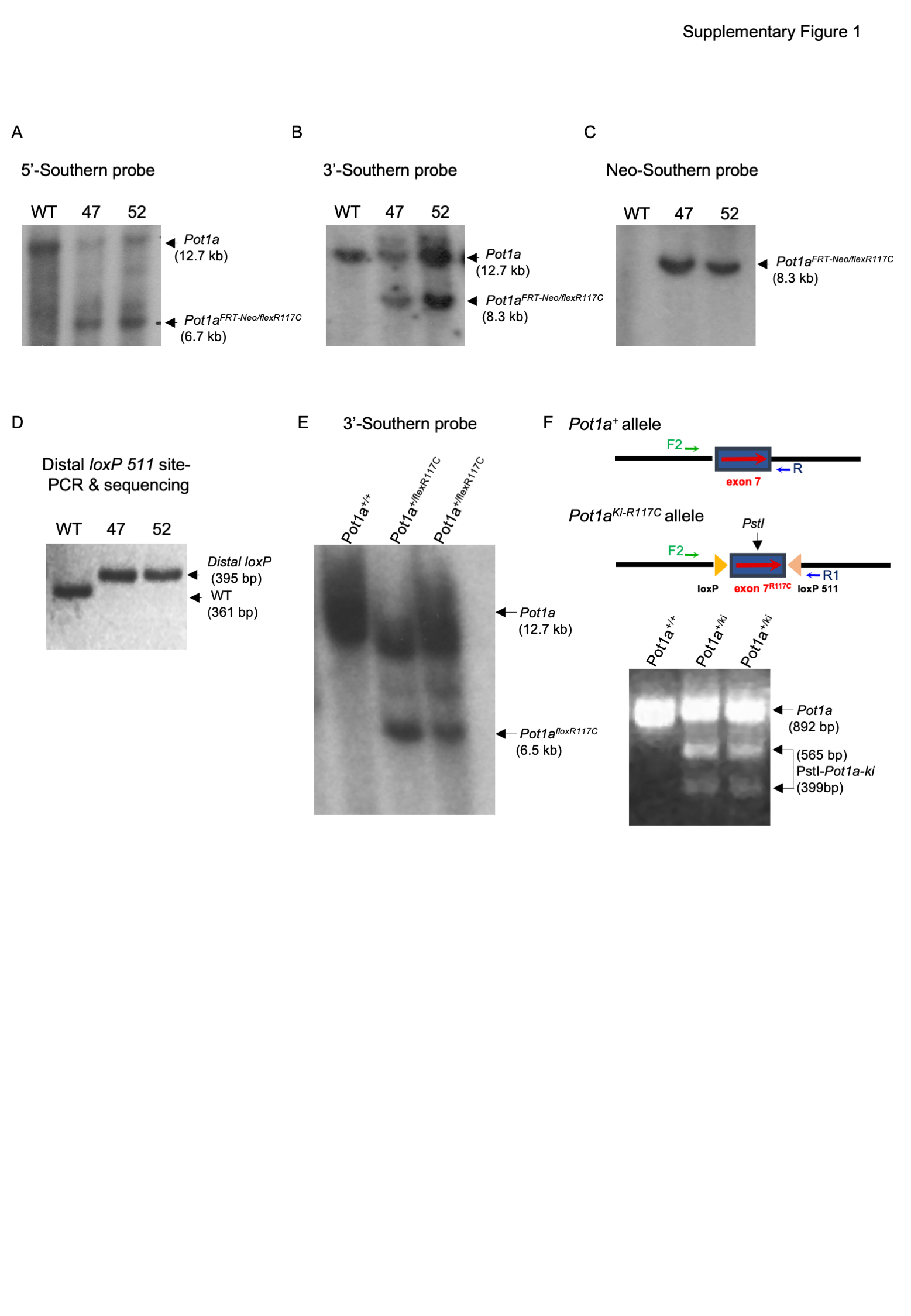

Supplement: S1 Fig — Genomic DNA was digested with EcoR1 restriction enzymes for southern analysis and the size of the fragments are indicated. A-C. Southern blot images from 5’- (A), 3’- (B) and neo (C) specific probes from two independent targeted stem cell clones (clone 47 and clone 52). D. Agarose gel of PCR product using primers F1 and R1 encompassing the distal LoxP511 site from the selected stem cell clones. These PCR products were further sequenced to assure the 3’-end of the targeted allele was not lost in the genome integration event. E. Southern blot image with the 3’-probe from EcoRI digested genomic DNA from mice after excision of the Neo cassette by the FLP recombinase. These mice were used as the parental mice for generating the Pot1a+/flex colonies. F. Schematic representation of the PCR genotyping reaction for the Pot1a+ and Pot1aki alleles. A representative image of an agarose gel showing the PstI cleaved PCR amplification products using primers upstream and downstream E7 (F2 and R1, respectively) from genomic DNA from Pot1a+/+ and Pot1a+/ki mice after cross with the EIIa-Cre tool mouse. (TIFF) [file pgen.1010260.s001.tiff]

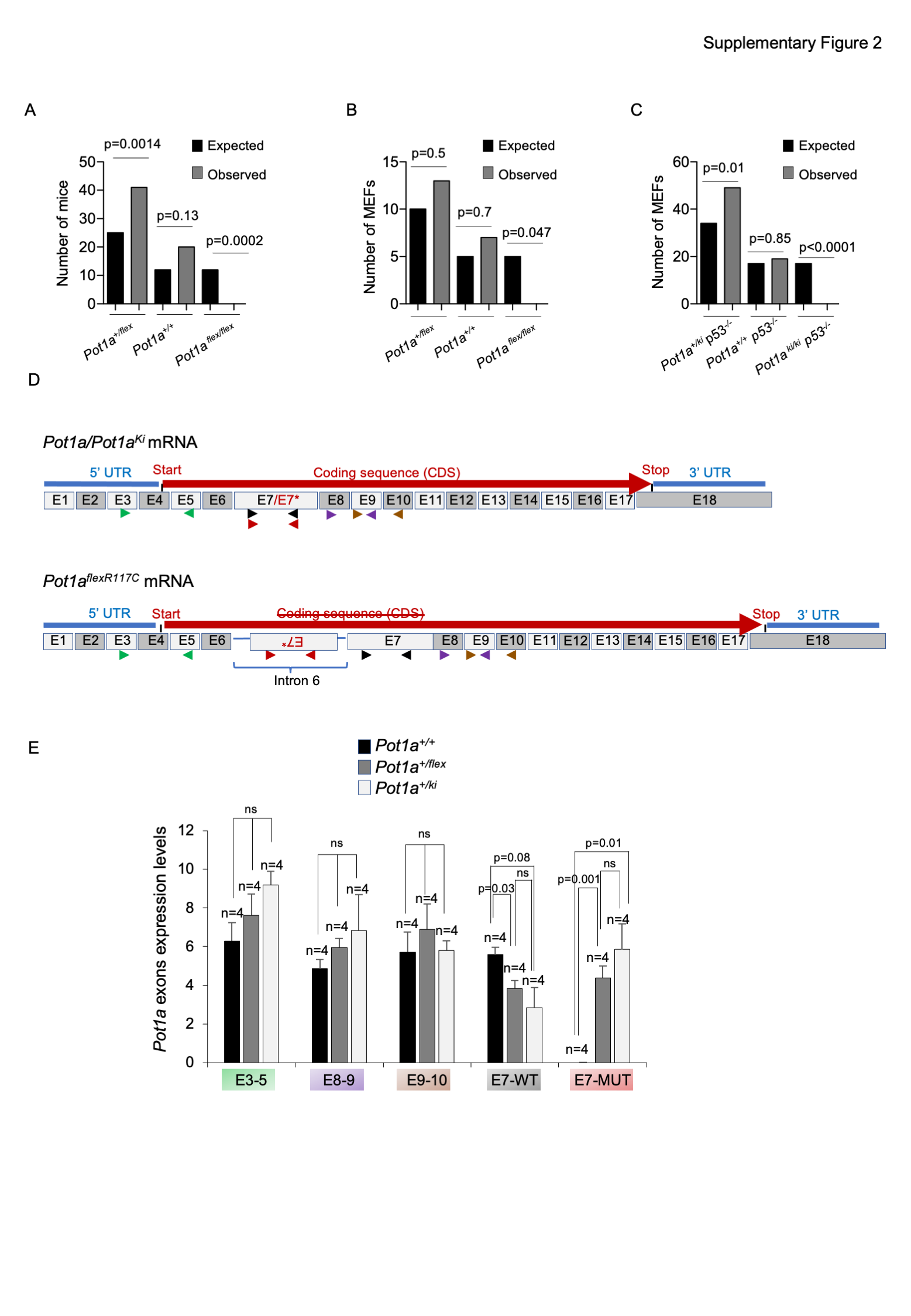

Supplement: S2 Fig — A. Expected and observed number of mice of the offspring from Pot1a+/flex intercrosses. B-C. Expected and observed number of embryos of the offspring from Pot1a+/flex (B) and from Pot1a+/kip53-/- (C) intercrosses. The Fisher’s exact test was used to determine statistical significance. p-values are indicated. D. Schematic representation of Pot1a, Pot1aki and Pot1aflex mRNA. The R117C substitution is within exon 7 (E7*). Five different primers pairs were used to quantify transcripts levels corresponding to E3-E5 (green arrows), E7 (black and red arrows for E7 wildtype and E7*, respectively), E8-E9 (purple arrows) and E9-E10 (brown arrows). The scheme is not drawn to scale. E. Quantification of expression levels by qRT-PCR of different exons in Pot1a, Pot1aki and Pot1aflex alleles in tail tissue of Pot1a+/+, Pot1a+/ki and Pot1a+/flex mice. A t-test two tailed was used for statistical analysis. The p-value is indicated. Mean values +/- SEM are represented. N = number of mice analyzed per genotype. (TIFF) [file pgen.1010260.s002.tiff]

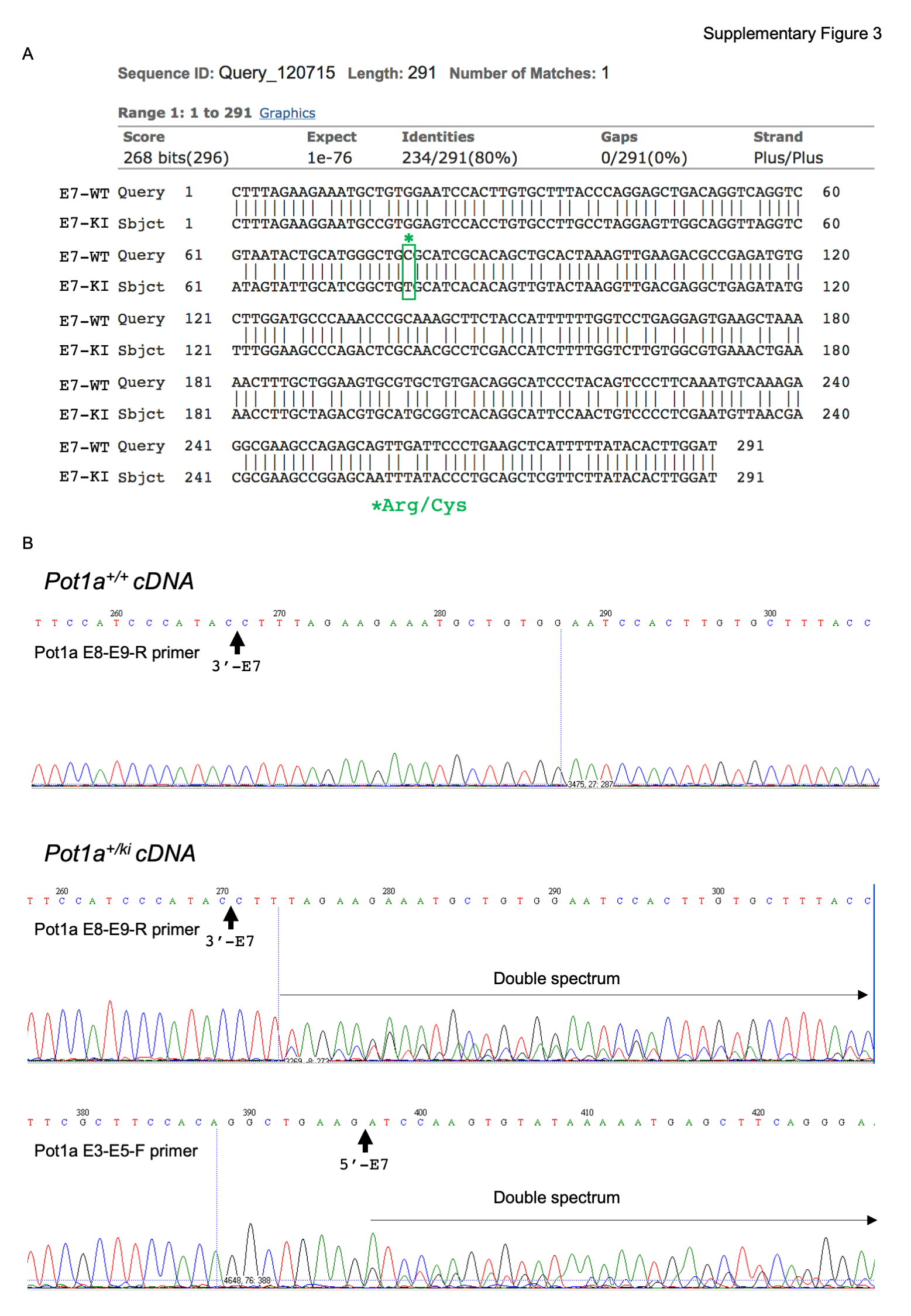

Supplement: S3 Fig — A. Nucleotide BLAST alignment of Pot1a wild type and mutant knock-in exon 7 sequences. The mutant exon 7 contains several silent substitutions and one missense mutation that results in an amino acid change from arginine to cysteine at position 117. B. Sequencing spectrum of wild type (upper panel) and mutant (lower panel) Pot1a-exon 7. The cDNA from Pot1a+/+ and Pot1a+/ki MEFs was PCR amplified using primers annealing within exon 3 (Pot1a E3-E5-F) and within exon9 (Pot1a E8-E9-R) (S1 Table). The PCR products were subjected to Sanger sequencing. The 5’ and 3’ ends of exon 7 and are indicated. (TIFF) [file pgen.1010260.s003.tiff]

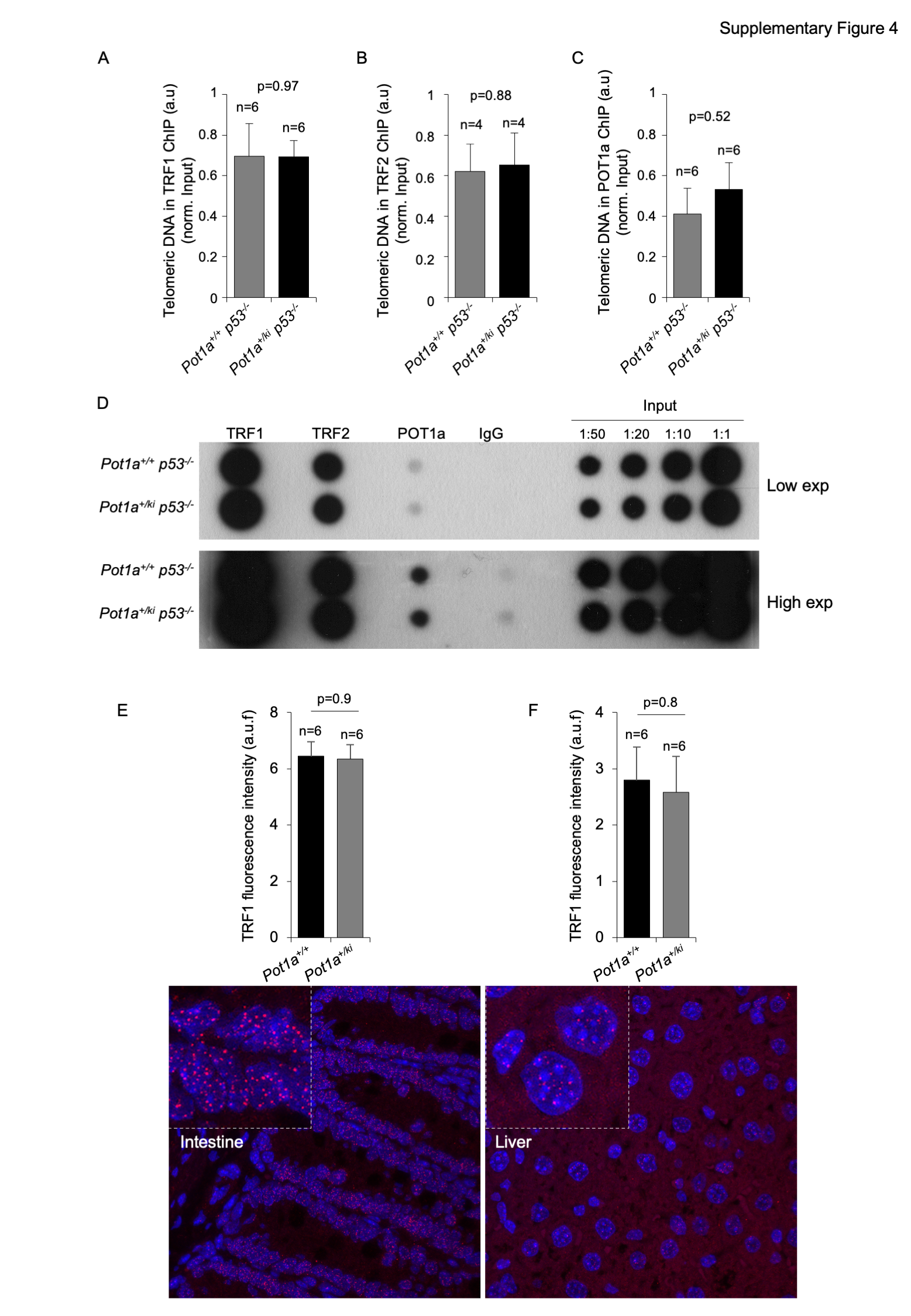

Supplement: S4 Fig — A-C. Quantification of telomeric DNA pulled down with anti-TRF1(A), with anti-TRF2 (B) and with anti-POT1a (C) of MEFs of the indicated genotype. DNA input signal is also shown. D. Representative images of chromatin immunoprecipitation (ChIP) of telomeric DNA. Low and high exposure images are shown. ChIP values are normalized by the input of each individual sample. E-F. Quantification of TRF1 fluorescence intensity levels in intestines (E) and in liver (F) of mice of the indicated genotype. Representative images of TRF1 immunofluorescence are shown below. Bars and error bars represent mean values ± SE. N = number of independent experiments. Student’s t-test was used for the statistical analysis. P-values are indicated. (TIFF) [file pgen.1010260.s004.tiff]

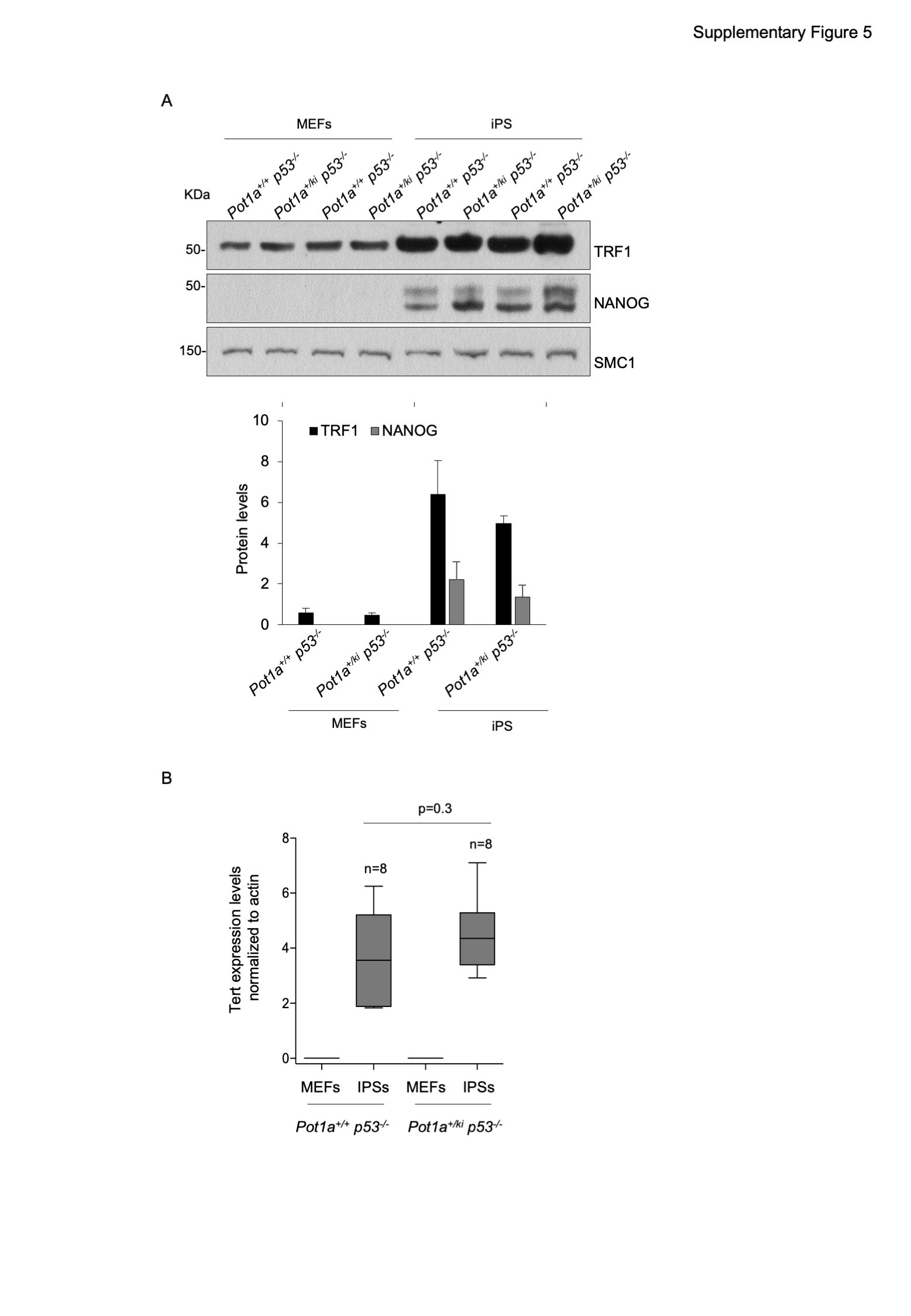

Supplement: S5 Fig — A. Quantification and representative western blot images of nuclear extracts of TRF1 and Nanog protein levels in Pot1a+/+p53-/- and Pot1a+/kip53-/- MEFs and iPS cells. SMC1 was used as loading control. IPS cells were generated from two independent MEFs from each genotype. The protein level quantification is represented in the bar plot. B. Quantification of Tert expression levels by q-RT-PCR in MEFs and IPs of the indicated genotype. Two MEFs and two iPS cells from each genotype were used for the analysis. The analysis was performed four times for each cell line. n = replicates. Two tailed Student’s t-test was used for the statistical analysis. P-values are indicated. (TIFF) [file pgen.1010260.s005.tiff]

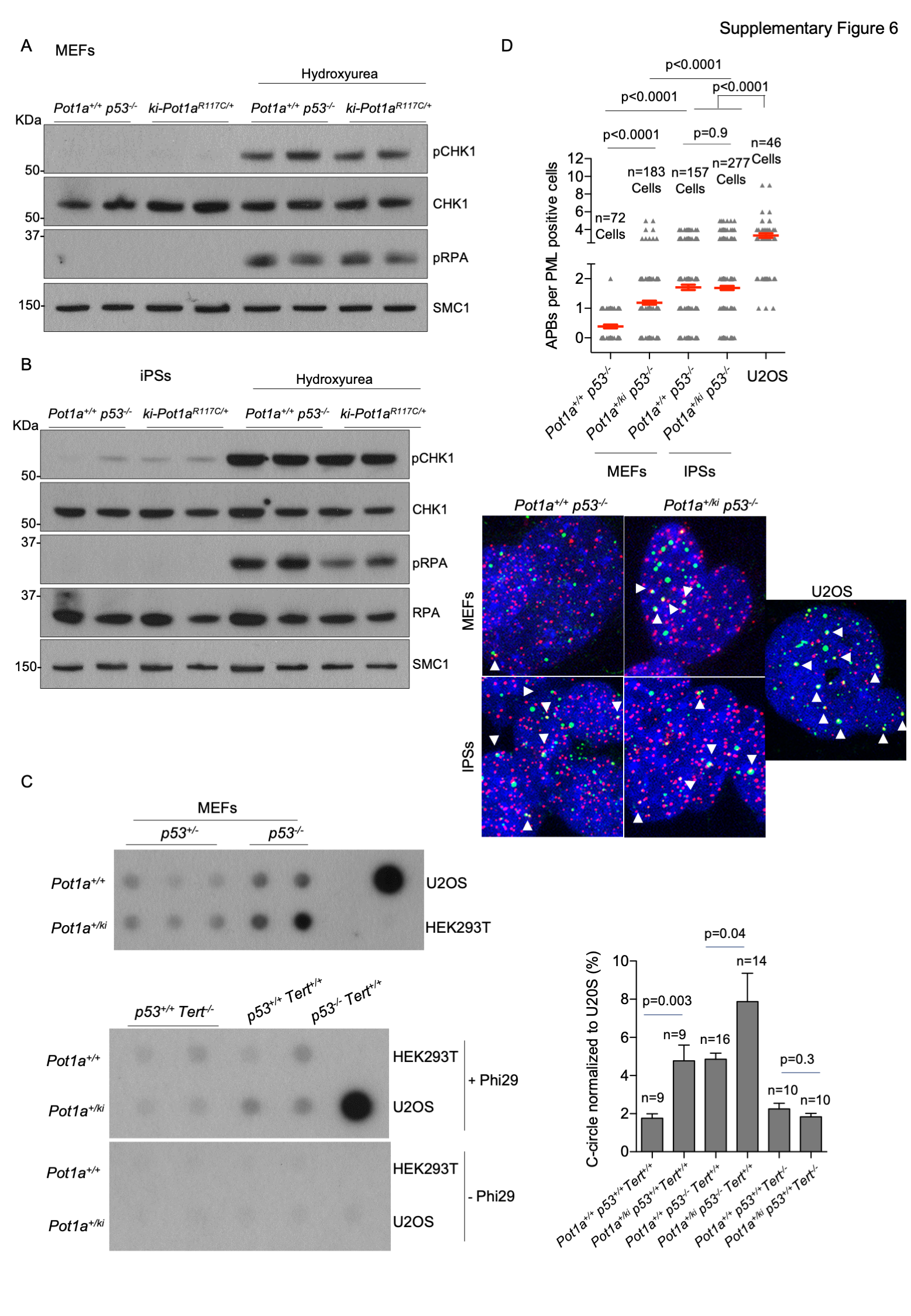

Supplement: S6 Fig — A,B. Representative western blot images of total cellular extracts of phosphor-CHK1, total CHK1, phospo-RPA, total RPA protein levels in Pot1a+/+p53-/- and Pot1a+/kip53-/- MEFs (A) and IPSs (B). SMC1 was used as loading control. Cells were treated with hydroxyurea (2 mM) for 3 hours as positive controls for replicative stress. C. C-circle quantification and representative dot-blot images of MEFs of the indicated genotype. U2OS and HEK293T cells were used as positive and negative controls, respectively. A negative control without Phi29 polymerase is also shown. The C-circle score is calculated as the percentage of the signal relative to that of ALT positive U2OS cell line. n = number of independent experiments D. Number of ALT-associated PML bodies (APBs) in PML positive cells (E) in MEFs of the indicated genotype. n = number of cells. Representative Immune-Fish images of PML and a telomeric probe. APBs were detected by PML and Telomere co-localizing foci (white arrowheads). A t-test two tailed was used for statistical analysis. Error bars represent standard error. The p-value is indicated. (TIFF) [file pgen.1010260.s006.tiff]

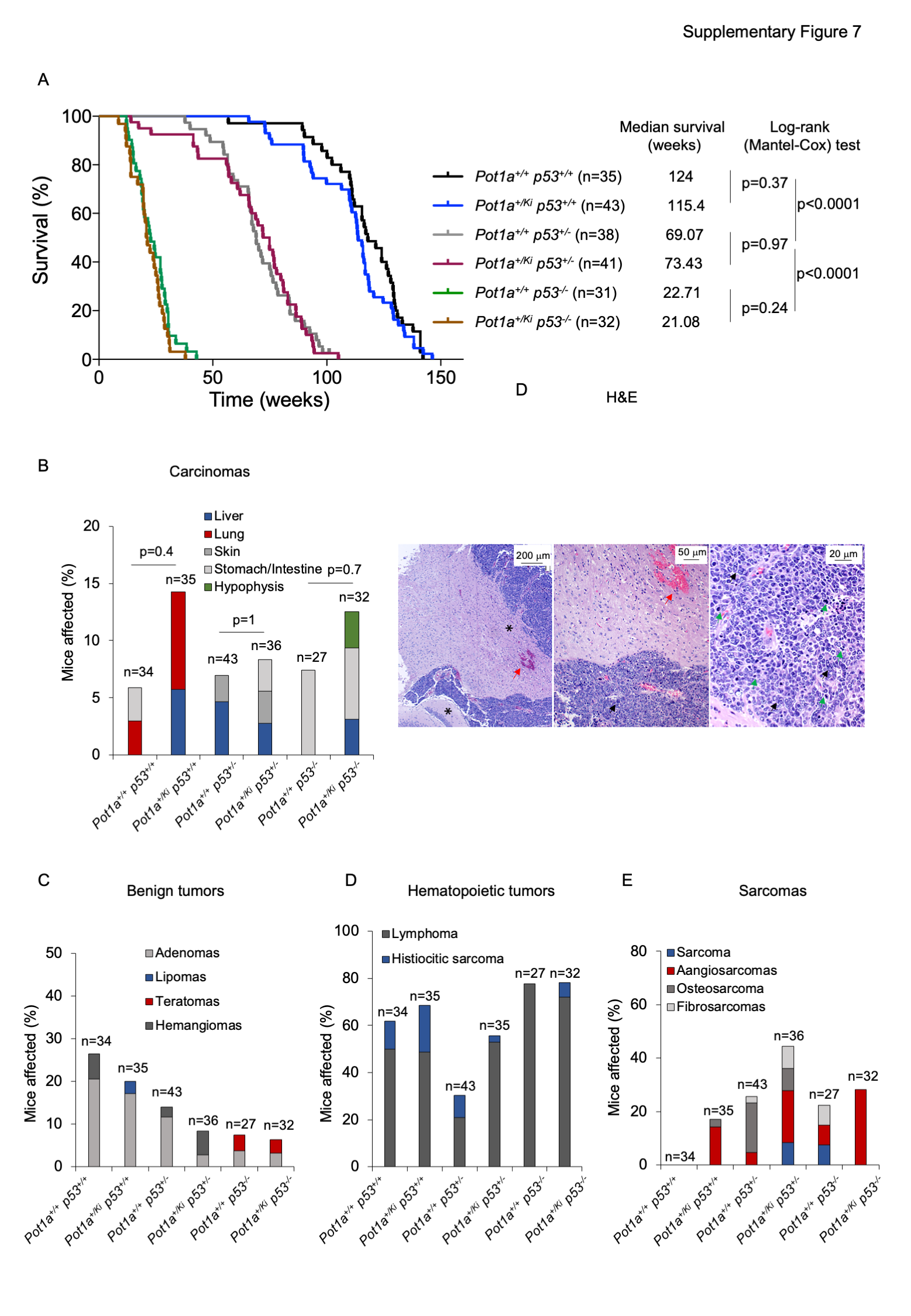

Supplement: S7 Fig — A. Kaplan-Meier survival curves of mouse cohorts of the indicated genotypes. Median survival values are indicated. A Log-rank (Mantel-Cox) test was performed for statistical analysis. P- values are indicated. n = number of mice. B. Percentage of mice presenting carcinomas at death. Representative H&E images of a hypophysis carcinoma are shown to the right. The neoplasm invades hypothalamus and the thalamus (asterisk). Hemorrhagic areas were present (red arrow). Atypical polygonal cells arranged in solid sheets with fibrovascular stroma. The tumor cells showed pleomorphic nuclei, large nucleoli (black arrow) and high number of mitosis (green arrows) were observed. Chi squared test was performed for statistical analysis. The number of mice and the p values are indicated. Scale bars are shown. C-E. Percentage of mice of the indicated genotype presenting benign (C), hematopoietic (D) and sarcoma (E) tumors at death. The types of tumors are indicated in the legend. Chi squared test was performed for statistical analysis. None of the comparisons were statistically significant (ns). N = number of mice. (TIFF) [file pgen.1010260.s007.tiff]

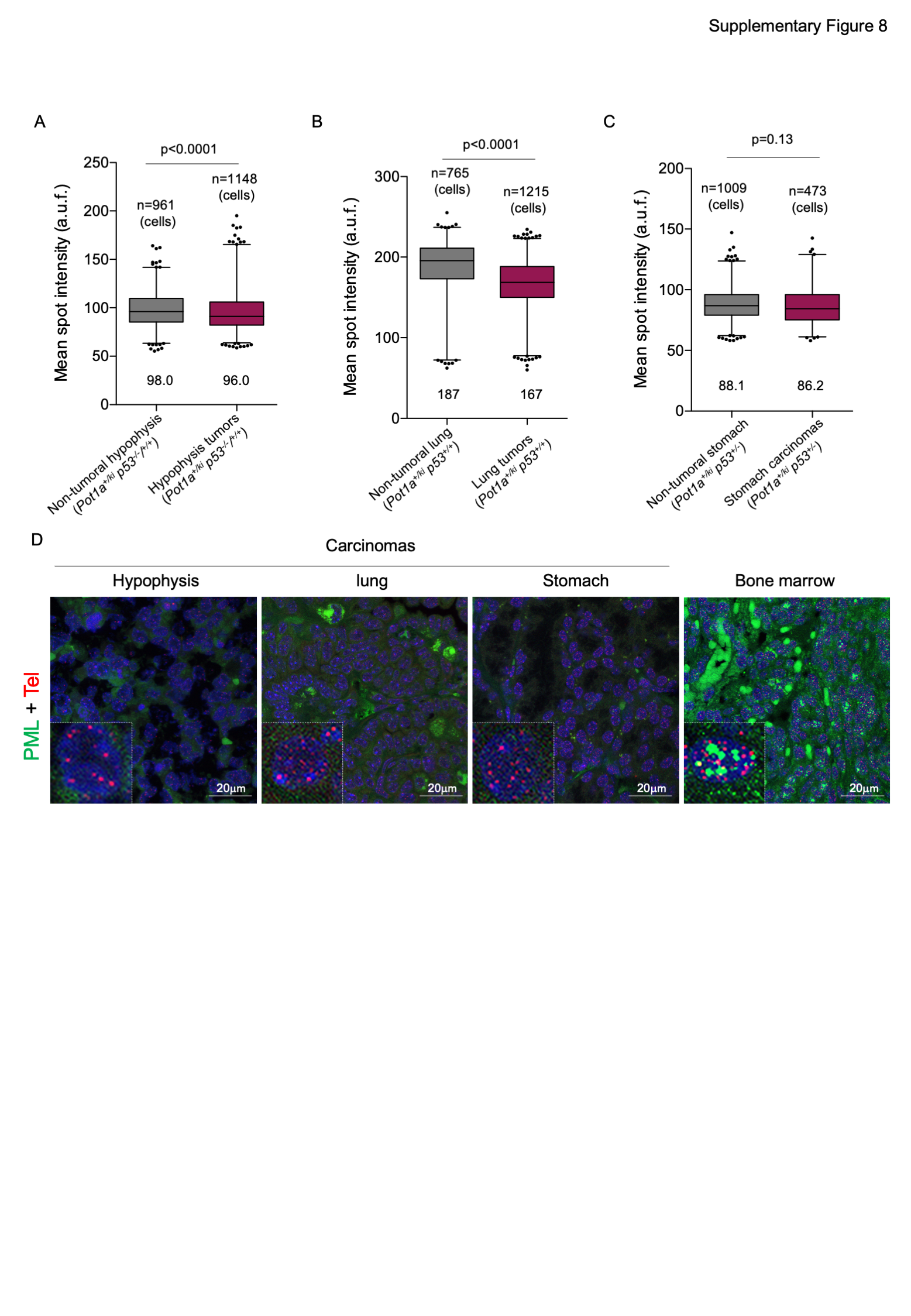

Supplement: S8 Fig — A. Telomere mean spot intensity in hypophysis tumors and in surrounding non-tumoral hypophysis tissue. Three adenomas from Pot1a+/kip53+/+ and one carcinoma from Pot1a+/kip53-/- mice at death were analyzed. B. Telomere mean spot intensity in lung tumors and in surrounding non-tumoral lung tissue. One lung adenoma and two bronchoalveolar carcinomas from Pot1a+/kip53+/+ mice at death were analyzed. C. Telomere mean spot intensity in stomach carcinomas and in surrounding non-tumoral stomach tissue. Two Pot1a+/ki/p53+/- stomach carcinomas were analyzed. The box-and-whisker graph shows the values lower and greater than first and 99th percentile for each group. Mean spot intensity is indicated in each case. n = number of cells. A t-test two tailed was used for statistical analysis. The p-values are indicated. D. Representative Immune-Fish images of PML and a telomeric probe in carcinomas from hypophysis, lung and stomach. Bone marrow sections were used as staining positive control. Insets correspond to higher magnification images. (TIFF) [file pgen.1010260.s008.tiff]

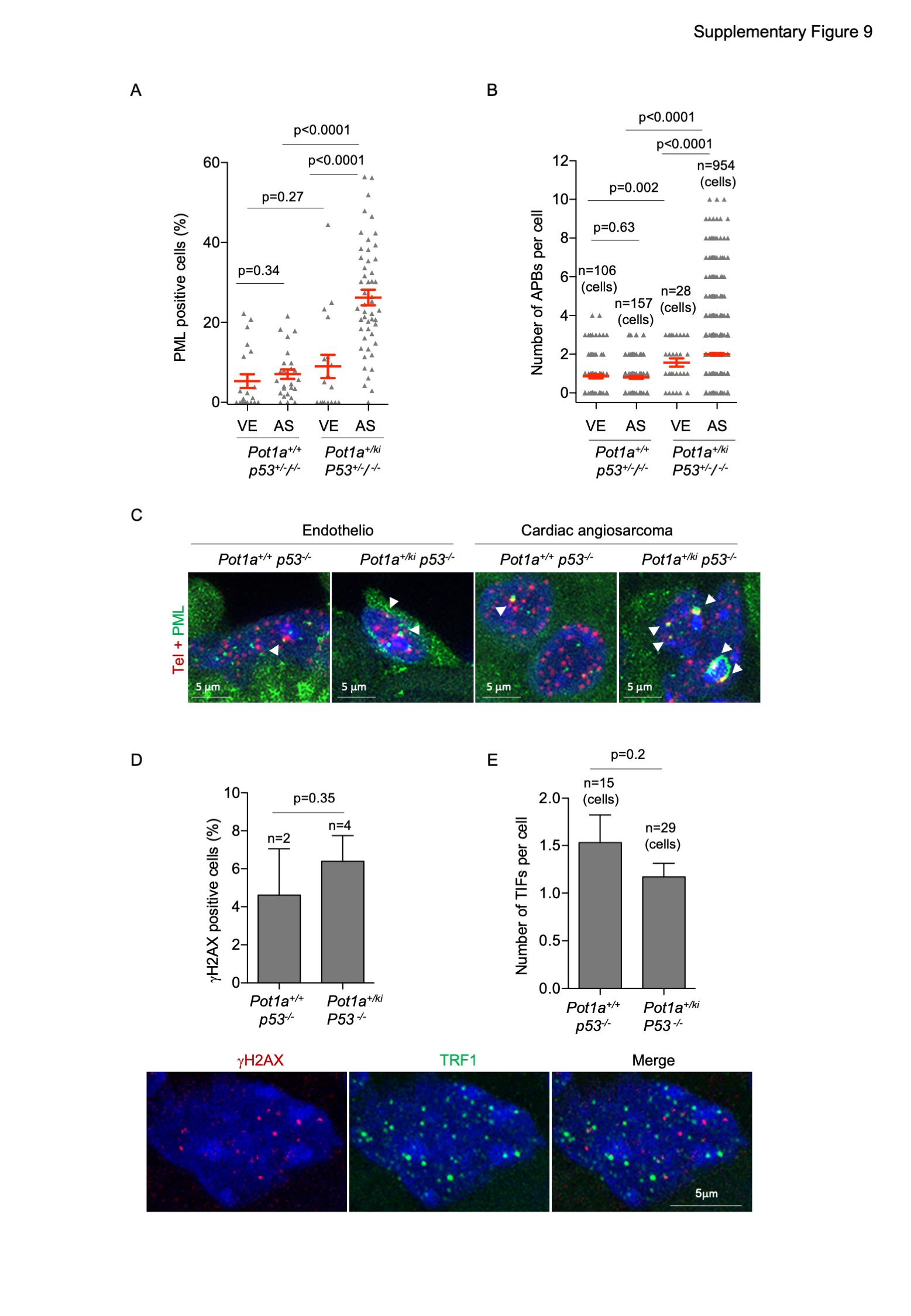

Supplement: S9 Fig — A-B. Percentage of PML positive cells (A) and number of ALT-associated PML bodies (APBs) per cell (B) in healthy vascular endothelium (VE) and in angiosarcomas (AS) from mice of the indicated genotype at death point. A PML positive cell was defined as having >2 foci. Two Pot1a+/+ tumors were analyzed, a utero angiosarcoma (Pot1a+/+p53+/-) and a cardiac angiosarcoma (Pot1a+/+p53-/-). Four Pot1a+/ki thoracic angiosarcomas were analyzed, three Pot1a+/kip53-/- and one Pot1a+/kip53+/-. Non-tumoral vascular endothelium from the same mice were also analyzed. F. Representative Immune-Fish images of PML and a telomeric probe in vascular endothelium and in cardiac angiosarcomas of the indicated genotypes. Vascular endothelium was identified by autofluorescence of red blood cells within the blood vessels. APBs were detected by PML and Telomere co-localizing foci (white arrowheads). D-E. Percentage of γH2AX positive cells (D) and number of Telomere-Induced Foci (TIF) per cell (E) in AS from mice of the indicated genotype at death point. The samples analyzed were the same as in A. A representative image of γH2AX and TRF1 staining is shown. TIFs were detected by γH2AX and TRF1 co-localizing foci. A t-test two tailed was used for statistical analysis. The p-values are indicated. (TIFF) [file pgen.1010260.s009.tiff]
