# Supplementary material for: A mouse model for Li-Fraumeni-Like Syndrome with cardiac angiosarcomas associated to POT1 mutations
Source: PLoS Genet. 2022 Jun 21;18(6):e1010260. doi: 10.1371/journal.pgen.1010260 (PMC9212151; doi:10.1371/journal.pgen.1010260)
Supplement: S1 Table — (DOCX) [file pgen.1010260.s010.docx]

**S1 Table 1:** Primers used in this study.

| **Name** | **sequence (5'-3')** |  |
| --- | --- | --- |
| Pot1a-F1 | CTGGGTAAAGCACAAGTGGATTC | |
| Pot1a-R1 | CCACTGTCCGACCTAGAATTGAC | |
| Pot1a-F2 | CACACTATTCCTTCACCTTAT | |
| q-Pot1a E9-E10-F | CTGGAGTTCCATCTCCACGG | |
| q-Pot1a E9-E10-R | TGCAGAGCTGTCCCCATTTT | |
| q-Pot1a E8-E9-F | TCAAGTGGCGAGATCCATCG | |
| q-Pot1a E8-E9-R | AAAACCCTAATGCCCCGACC | |
| q-Pot1a-E3-E5-F | TCGGTTGTGGAAAGCCTACA | |
| q-Pot1a-E3-E5-R | GGGAGCTGTTGAAACCAAAGAC | |
| q-Pot1a-E7-WT-F | TGGCTTCGCCTCTTTGACA | |
| q-Pot1a-E7-WT-R | CATCGCACAGCTGCACTAAAG | |
| q-Pot1a-E7-Ki-F | CGCGTCGTTAACATTCGAGG | |
| q-Pot1a-E7-Ki-R | GGCTGTGCATCACACAGTTG | |
| q-Pot1a-E17-E18-F | TCCCAGCATCTGAAGTCCTC | |
| q-Pot1a-E17-E18-R | AATTCGTCGCTCTGTTCCGA | |
| q-TERT-F | GGATTGCCACTGGCTCCG | |
| q-TERT-R | TGCCTGACCTCCTCTTGTGAC | |
